# Lipoic Acid‐Intervened Decellularized Stem Cell Spheroid‐Based Injectable Granular Gel for Diabetic Tissue Regeneration

**DOI:** 10.1002/advs.202521924

**Published:** 2026-04-07

**Authors:** Tao Wang, Haowei Fang, Lili Qi, Song Myoungseop, Aawrish Khan, Lunli Gong, Guangdong Zhou, Kunxi Zhang, Haiyan Cui

**Affiliations:** ^1^ Department of Plastic and Cosmetic Surgery Tongji Hospital School of Medicine Tongji University Shanghai P. R. China; ^2^ Institute of Aesthetic Plastic Surgery and Medicine School of Medicine Tongji University Shanghai P. R. China; ^3^ Department of Polymer Materials School of Materials Science and Engineering Shanghai University Shanghai P. R China; ^4^ Department of Plastic and Reconstructive Surgery Shanghai Ninth People's Hospital Shanghai Key Laboratory of Tissue Engineering Shanghai Jiao Tong University School of Medicine Shanghai P. R. China

**Keywords:** decellularized matrix, granular gel, lipoic acid, stem cell spheroid, tissue engineering

## Abstract

Advancements in tissue engineering have revolutionized therapeutic paradigms for diabetic tissue defects; however, the lack of applicable scaffold containing various bioactive substance aggregates remained a critical bottleneck hindering satisfactory repair effect. In this study, adipose‐derived stem cells (ADSCs) were functionally re‐engineered using lipoic acid (LA) to fabricate a novel LA‐intervened stem cell spheroid (LA‐SCS) with enhanced paracrine activity and extracellular matrix (ECM) biosynthetic capacity. Subsequent decellularization mitigated immunogenicity, yielding LA‐intervened decellularized stem cell spheroid (LA‐dSCS). In vitro assays confirmed its immunomodulatory potency, as evidenced by the activation of signaling cascades associated with macrophage reprogramming, homeostasis, and autophagy. Furthermore, leveraging the intrinsic viscoelastic properties of the LA‐dSCS, a convenient preparation method for preparing LA‐dSCS derived injectable material was established, wherein LA‐dSCS micro‐particles assemble into LA‐dSCS granular gel. In vivo studies using diabetic rat models demonstrated closure of both wound and cranial defects. Collectively, this study established a biomimetic engineering strategy that integrates cell‐free bioactive aggregates with injectable granular gels, offering a novel proof‑of‑concept strategy for the regeneration of complex diabetic tissue defects.

## Graphical abstract

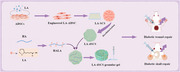

## 1 Introduction

Diabetes mellitus is a multifactorial metabolic disorder that poses a critical threat to global human health [1, 2, 3]. Tissue defects (e.g., skin and bone) in diabetic subjects often exhibit prolonged non‐healing, primarily attributed to excessive local microenvironmental inflammation and dysregulated bone cell functions (osteoblasts and osteoclasts) [4, 5, 6, 7]. Specifically, the failure of macrophages to transition from pro‐inflammatory M1 phenotype to anti‐inflammatory repair‐promoting M2 phenotype disrupts the inflammatory‐to‐proliferative phase transition, thereby impeding healing [8, 9]. Current clinical interventions, including debridement with dressing changes, negative pressure wound therapy, and autologous bone grafting, suffer from inherent limitations such as extended treatment durations, insufficient microenvironmental modulation, high surgical risks, and donor‐site morbidity [10].

In recent years, tissue engineering strategies based on stem cells have garnered growing attention [11, 12]. As a pivotal type of stem cell, ADSCs have emerged as one of the preferred cellular sources owing to their ease of access and inherent immunomodulatory properties [13]. Stem cells exert immunomodulatory effects via paracrine secretion of bioactive factors such as basic fibroblast growth factor (bFGF), vascular endothelial growth factor (VEGF), and hepatocyte growth factor (HGF) [14, 15]. A body of studies, including our previous studies, have demonstrated that 3D biomimetic culture of stem cell spheroids can recapitulate the in vivo 3D aggregated microenvironment of cells (e.g., intercellular interactions within tissues and nutrient gradients), exhibiting stronger immunomodulatory capacity, cell viability, paracrine activity, and ECM production ability compared to 2D culture [16, 17, 18]. The preparation and application of stem cell spheroids in tissue regeneration are thus of great value. However, direct transplantation of either ADSCs or stem cell spheroids into the diabetic microenvironment is prone to induce apoptosis and necrosis. The release of intracellular contents following cell membrane rupture exacerbates local inflammation, forming a vicious cycle that impairs their efficacy in regenerating diabetes‐related tissues [19]. Additionally, it entails potential risks such as immune rejection, tumorigenicity, and disease transmission. Thus, how to circumvent these issues while retaining their immunomodulatory capacity has become an urgent problem to be addressed.

Notably, the ECM components secreted by stem cell spheroids are more akin to the composition of natural in vivo ECM, encompassing structural components such as collagens (types I, III, etc.), glycosaminoglycans (hyaluronic acid, chondroitin sulfate, etc.), fibronectin, and laminin [20]. Concurrently, they are enriched with stem cell‐derived paracrine growth factors (e.g., VEGF, EGF), cytokines (e.g., IL‐6, TGF‐β), and other bioactive components, including exosomes [21, 22]. If we can prepare functional‐enhanced ECM through preprogramming, with cellular immune components removed from the cell spheroids, it is anticipated to mitigate multiple potential risks associated with the direct application of stem cell spheroids and achieve better tissue repair efficacy in diabetic hosts. To achieve this objective, it is essential to establish intervention strategies targeting the diabetes microenvironment and the preparation approach of decellularization matrix‐based biomaterials.

First, drug or molecular induction represent a key approach to achieve targeted regulation of stem cell pre‐programming, which can modulate cellular functions such as paracrine activity and metabolism, thereby altering ECM components [23, 24, 25]. LA, an essential mitochondrial coenzyme for aerobic metabolism, possesses high biocompatibility and antioxidant activity [26]. It can scavenge superoxide and peroxide free radicals, and exhibit remarkable performance in metabolic reprogramming and anti‐oxidative stress; notably, it has been approved by the FDA for clinical treatment of diabetes peripheral neuropathy [27, 28, 29, 30]. Nevertheless, the content of ECM bioactive factors and the enhanced immunoregulatory effects of LA following co‐culture with ADSCs under both 2D and 3D conditions remain unelucidated.

Second, similar to the article we have previously published [31], decellularization technology can reduce the risks of immune rejection, tumorigenicity, and disease transmission associated with direct application, while maximizing the retention of the 3D structure of secreted ECM and bioactive molecules (e.g., growth factors, cytokines) within it, thereby facilitating microenvironment remodeling during regeneration and repair of diabetes‐related tissue defects [31, 32]. However, the challenge of fabricating decellularized spheroid matrices into functional tissue repair materials remains an urgent issue to be addressed. Currently, numerous studies employ chemical or physical modification, followed by digestion and dissolution to produce gels [33, 34]. Unfortunately, this approach results in disruption and loss of bioactive components [35, 36]. Thus, there is an imperative need to establish a convenient and efficient strategy for converting decellularized spheroid matrices into materials for tissue filling. Granular gels, as bulk hydrogels formed by dense assembly of micro‐particles, possess inherent advantages such as micrometer‐scale porosity and injectability [37, 38]. By assembling decellularized spheroid matrix micro‐particles into granular gels, it may be feasible to prepare injectable tissue repair materials while maximizing the retention of bioactive components within the decellularized matrix.

**FIGURE 1:**
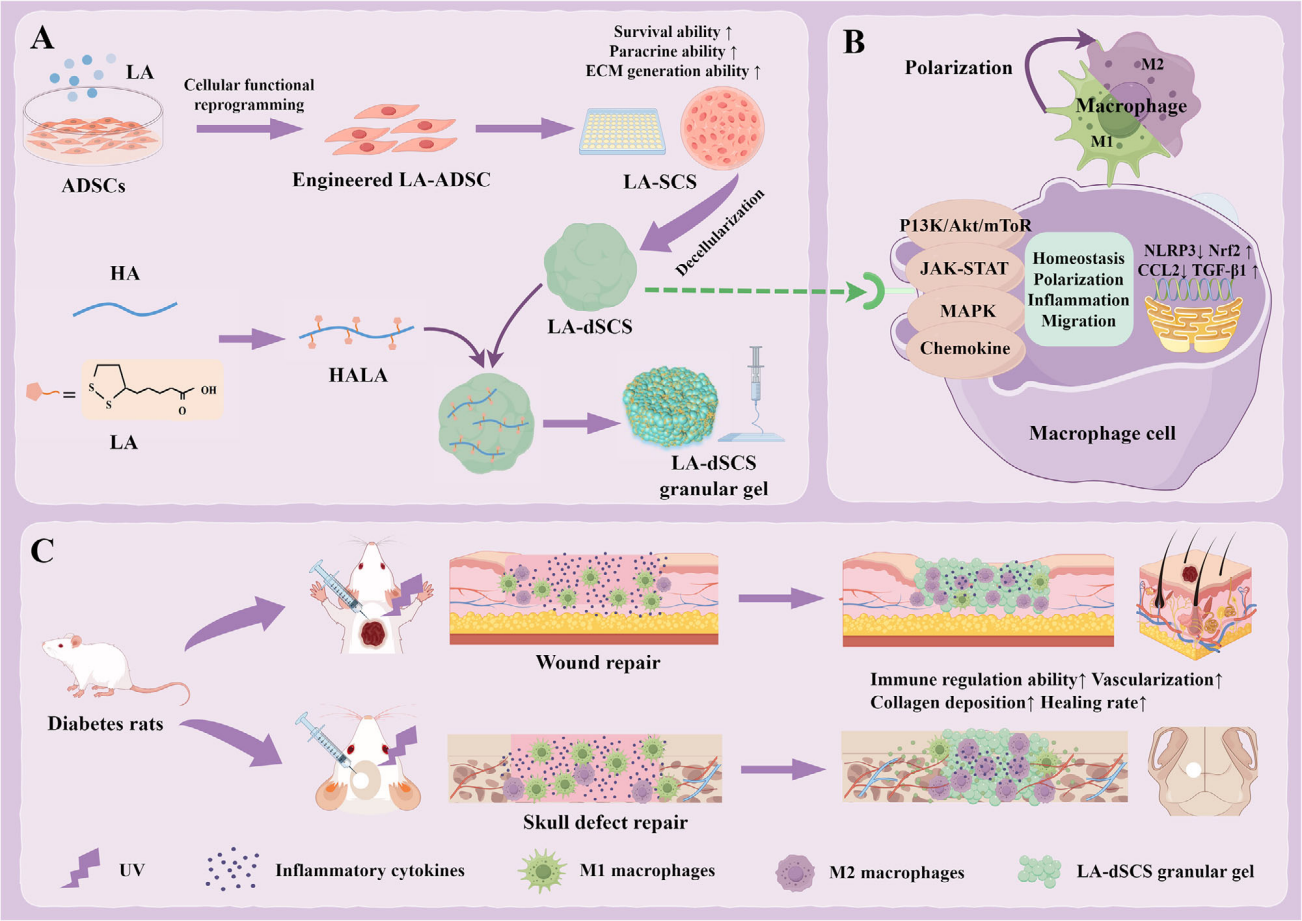
Schematic illustration of construction of LA‐dSCS granular gel for tissue engineering of diabetic wound and skull defects. (A) Construction of LA‐dSCS granular gel. (B) Schematic illustration of the mechanism of LA‐dSCS in macrophage immune regulation process. (C) Schematic illustration of the application of LA‐dSCS granular gel in diabetic wound and skull defect tissue engineering.

In this study, we utilized LA to reprogram ADSCs functions, thereby prepared LA‐intervened stem cell spheroid (LA‐SCS) with enhanced paracrine/EC production capacity. Following decellularization, LA‐intervened decellularized stem cell spheroids (LA‐dSCS) were obtained to retain their bioactive components. In vitro experiments confirmed that LA‐dSCS possess immunomodulatory ability and can activate signaling pathways regulating macrophage reprogramming, homeostasis, and autophagy. To address the limitations of standalone LA‐dSCS application, LA was grafted onto hyaluronic acid (HA) to synthesize LA‐grafted HA (HALA) solution, which was then pre‐assembled with LA‐dSCS to fabricate injectable LA‐dSCS granular hydrogels. These hydrogels were subsequently injected into a diabetic wound and skull defect. In vivo experiments demonstrated optimal defect closure, microenvironment remodeling, collagen deposition, and vascularization. In summary, this study constructed a novel stem cell‐derived cell‐free bioactive aggregate (LA‐dSCS) and formed injectable photosensitive LA‐dSCS granular gels via pre‐assembly with HALA. This research offered a novel proof‑of‑concept strategy into the development of biomaterials targeting multiple tissue defects in diabetes (Figure 1).

## 2 Results and Discussion

### 2.1 Preparation and Biological Evaluation of LA‐Intervened Stem Cell Spheroid (LA‐SCS)

#### 2.1.1 Biocompatibility Assessment of LA and LA Co‐Culture

**FIGURE 2:**
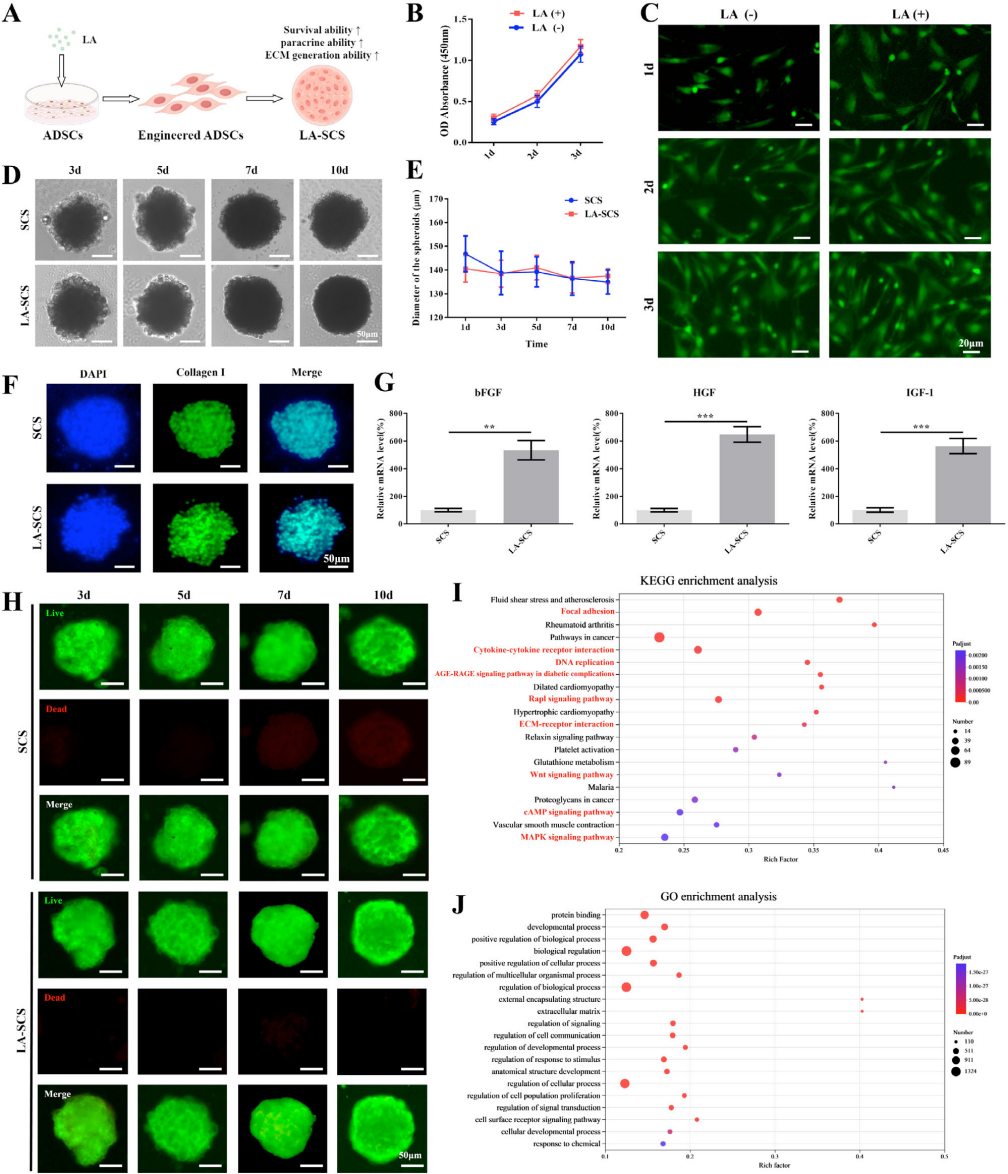
Preparation and biological evaluation of LA‐SCS. (A) Schematic illustration of LA‐SCS fabrication. (B) CCK‐8 assay for LA biocompatibility evaluation. (C) Live/dead staining assessment of LA biocompatibility, confirming low cytotoxicity. (D) Macroscopic morphology of cell spheroids, showing no significant morphological disparity between groups. (E) Quantitative analysis of cell spheroid diameter. (F) COL I immunostaining of cell spheroids, demonstrating higher collagen expression in the LA‐SCS group. (G) qRT‐PCR analysis of paracrine gene expression, indicating enhanced expression in the LA‐SCS group. (H) Live/dead staining of cell spheroids, revealing better viability in the LA‐SCS group. (I) KEGG pathway enrichment analysis from RNA‐seq of cell spheroids. (J) GO functional enrichment analysis from RNA‐seq of cell spheroids. Independent sample t test (G) was used for statistical analysis; *n* = 5 for B, E, and G, *n* = 3 for I and J; All data were depicted as means ± SD; ^**^ *p* < 0.01, ^***^ *p* < 0.001. [Correction added on 23 April 2026, after first online publication: Figure 2D, SCS, 7d, image corrected.]

Superior biocompatibility is a crucial factor for the successful implementation of the intervention [39]. The current results showed that LA (+) group did not significantly inhibit cell proliferation (Figure 2B), with minimal dead cells observed (Figure 2C). HE staining (Figure S1) revealed normal spindle‐shaped morphology in LA (+) group, while phalloidin‐FITC staining indicated enhanced actin cytoskeleton organization, forming a dense network conducive to cytoskeletal remodeling. TUNEL assay showed no significant apoptotic cells, confirming maintenance of apoptosis homeostasis.

LA was co‐cultured with adherent ADSCs in petri dishes to generate engineered ADSCs with enhanced functionality, circumventing the limitations of direct LA intervention in cell spheroids. NANOG, OCT4, and SOX2 were key regulators of stem cell self‐renewal, proliferation, paracrine function, and multi‐directional differentiation potential [40, 41]. Immunofluorescence showed higher expression of three markers in LA‐ADSC group under a diabetic microenvironment (Figure S2), indicating LA‐preserved stemness. qRT‐PCR results (Figure S3) were consistent. TNF‐α immunostaining and gene expression (Figures S4 and S5) revealed reduced pro‐inflammatory signals in LA‐ADSC, confirming LA‐enhanced anti‐inflammatory capacity.

#### 2.1.2 Cell Spheroid Fabrication and Characterization

The results of 3D culture showed that SCS and LA‐SCS groups showed incomplete aggregation with similar morphology at day 3 post‐seeding. By day 5, most cells aggregated; by days 7–10, LA‐SCS group spheroids exhibited more regular, plump, and compact structures, indicating cell‐cell adhesion (Figure 2D). Diameter analysis (Figure 2E) showed initial enlargement due to incomplete aggregation, followed by shrinkage. LA‐SCS group had slightly larger diameters at days 7–10, though not statistically significant.

COL I immunofluorescence (Figure 2F) revealed stronger signals in LA‐SCS group, indicating enhanced ECM production. Paracrine gene expression and ELISA assay (bFGF, HGF, IGF‐1) were significantly higher in LA‐SCS group (Figure 2G; Figure S6). Live/dead staining (Figure 2H) in normal microenvironment showed viable cells in both groups at days 3 and 5. By day 7, scattered dead cells appeared in SCS group, likely due to nutrient depletion and inflammatory accumulation, with increasing death by day 10. In contrast, LA‐SCS showed minimal dead cells throughout culture, suggesting LA‐improved hypoxia resistance and metabolic regulation.

To dissect the gene expression profiles and unravel the mechanistic basis for phenotypic differences between the two groups under 3D culture conditions, RNA‐seq transcriptomic analysis was performed. The current results indicate 2689 differentially expressed genes (1013 upregulated, 1676 downregulated) between groups (Figure S7). KEGG/GO enrichment (Figure 2I,J) revealed pathways related to cell function, external stimuli, signal transduction, ECM, and inflammation. Functional annotation analysis demonstrated that differentially expressed genes were broadly distributed across major categories including metabolism, environmental information processing, cellular metabolism, and cell growth (Figure S8). Furthermore, we performed Western Blot analysis focusing on key molecules in the Wnt, AGE‐RAGE, and MAPK signaling pathways, which further confirmed the RNA‐seq results (Figure S9).

Among these, the AGE‐RAGE signaling pathway in diabetic complications was intimately linked to inflammatory responses [42]. Binding of advanced glycation end products (AGEs) to their receptor (RAGE) activates NADPH oxidase, triggering a ROS burst that exacerbates cellular inflammation and apoptosis. The LA‐SCS group potentially down‐regulated this pathway to inhibit its activation, suppressing RAGE expression and excessive MAPK (p38/JNK) signaling to promote cell survival. Moreover, enrichment of cytokine‐cytokine receptor interaction, focal adhesion, and ECM pathways indicated enhanced crosstalk among cells, cytokines, and ECM in LA‐SCS group, contributing to cytoskeletal stability and cell adhesion. This facilitated bidirectional regulation between cytokine secretion and cellular responses, while ECM provided protective effects, establishing a positive feedback loop. KEGG analysis further revealed significant enrichment of the DNA replication pathway, alongside GO biological processes including developmental progression, cell proliferation regulation, and cellular process activation. These findings suggested targeting of the Wnt signaling pathway and DNA replication‐related cascades to maintain cellular proliferative potential during cell cycle regulation [43].

**FIGURE 3:**
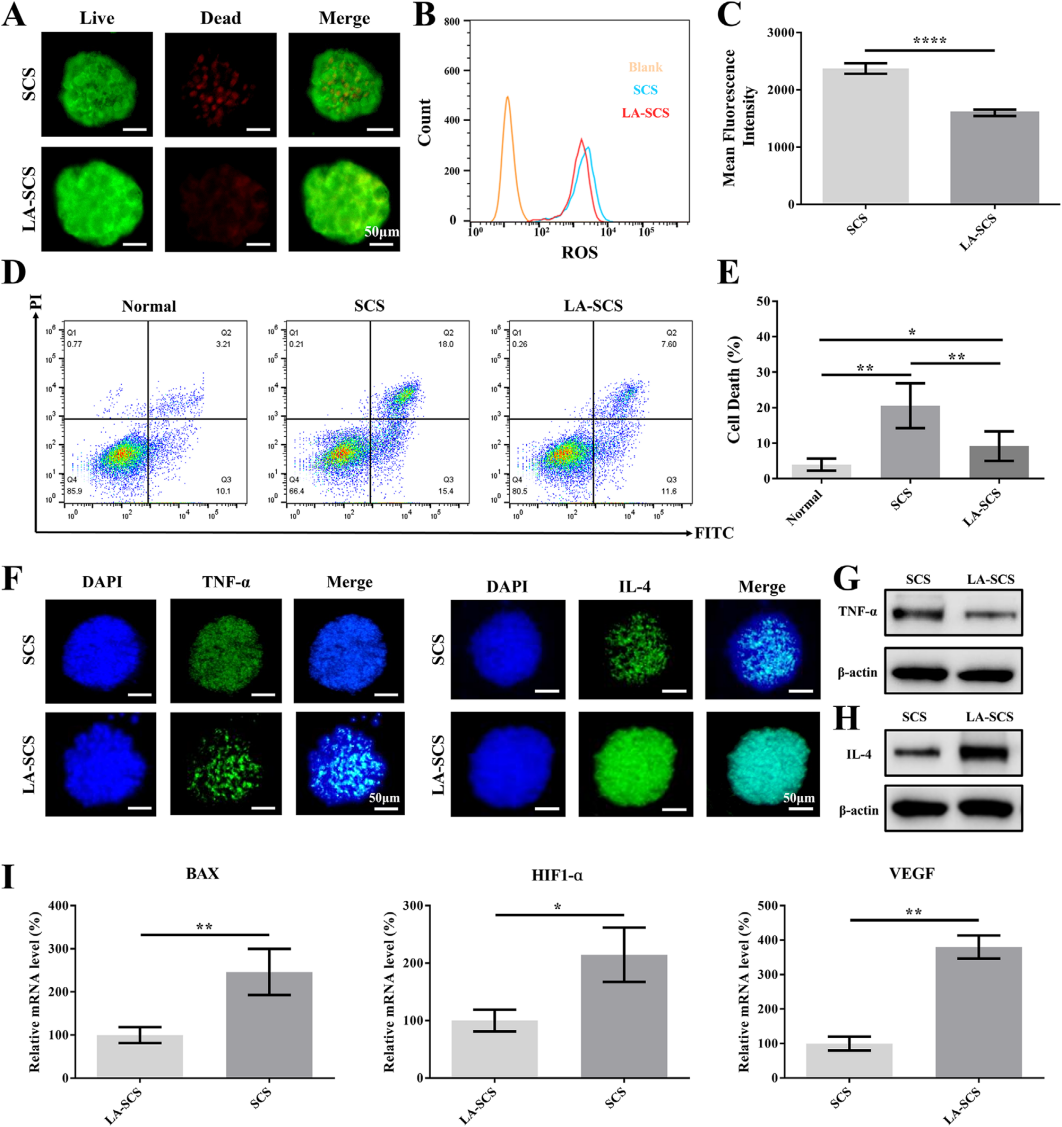
Biological assessment of LA‐SCS cell spheroids in the diabetic microenvironment. (A) Live/dead staining demonstrated better viability in the LA‐SCS group. (B) Flow cytometric analysis of ROS. (C) Quantitative ROS flow cytometry confirmed enhanced antioxidant capacity in the LA‐SCS group. (D) Flow cytometric apoptosis analysis showed the lowest apoptosis rate in the LA‐SCS group. (E) Quantitative analysis of flow cytometric apoptosis results. (F) Immunofluorescence assessment of pro‐inflammatory and anti‐inflammatory markers. (G) Western blot analysis of IL‐4 protein expression. (H) Western blot analysis of TNF‐α protein expression. (I) Gene expression evaluation of BAX, HIF‐1α, and VEGF. Independent sample t test (C and I) and one‐way analysis of variance (E) were used for statistical analysis; *n* = 5; All data were depicted as means ± SD; ^*^ *p* < 0.05, ^**^ *p* < 0.01.

Furthermore, we evaluated the cell spheroids in a diabetic microenvironment. As shown in Figure 3A, SCS group exhibited significantly stronger red fluorescence intensity (indicative of dead cells) with aggregated necrotic foci. ROS quantification and average fluorescence intensity confirmed higher oxidative stress in SCS group compared to LA‐SCS group (Figure 3B,C). This suggested that high glucose and inflammatory factors in diabetic microenvironments induce oxidative stress, leading to ROS‐mediated damage to cellular macromolecules (DNA/proteins/lipids), impairing normal functions and triggering cell death.

As shown in Figure 3D (flow cytometric apoptosis analysis), the Normal group showed 85.9% viable cells (Q4 quadrant), while SCS group exhibited a 20% reduction (66.4% viability). Notably, LA‐SCS group maintained 80.5% viability, approaching the Normal group levels, demonstrating enhanced antioxidant capacity against diabetic stress. Immunofluorescence (IL‐4/TNF‐α) and western blot analyses (Figure 3F–H) revealed higher IL‐4 and lower TNF‐α expression in LA‐SCS group, confirming better anti‐inflammatory potential. Gene expression profiling showed that LA‐SCS group has downregulated BAX (apoptosis) and HIF‐1α (hypoxia), with upregulated VEGF (angiogenesis) (Figure 3I). The above results further confirmed that the LA‐SCS group had better anti‐oxidative stress ability in the oxidative stress environment mimicking diabetes, and initially explained the reason for the better survival ability in flow analysis.

### 2.2 Fabrication and Biological Characterization of LA‐Intervened Decellularized Stem Cell Spheroids (LA‐dSCS)

#### 2.2.1 Decellularization Evaluation

Diabetic tissue defect regions frequently exhibit pathological hallmarks, including hyperglycemia, oxidative stress, chronic inflammation, and inadequate blood supply [4]. Such a microenvironment can markedly impair the survival, proliferation, and functional performance of viable stem cells (e.g., their capacity for cytokine secretion) [44]. Moreover, living stem cell spheroids express cell‐specific antigens (such as MHC molecules) on their surface, which were prone to inducing host immune rejection responses, thereby triggering cellular inactivation [19, 45]. Additionally, under conditions of immune dysfunction in diabetic patients, these cells may undergo aberrant differentiation toward abnormal phenotypes (e.g., excessive adipogenesis) and even carry a potential risk of tumorigenesis [46, 47].

Decellularized treatment can eliminate the viable cellular components that were susceptible to microenvironmental perturbations, retaining solely the ECM secreted by the cell spheroids. As a natural “biological scaffold,” this retained ECM not only possessed physical structural stability to resist high glucose‐induced matrix degradation but also preserved components such as collagen, glycosaminoglycans, and non‐collagenous glycoproteins [31, 32, 48]. While remodeling the diabetic microenvironment, it provided adhesion sites and migration pathways for host endogenous repair cells (e.g., fibroblasts and vascular endothelial cells), thereby circumventing issues such as immune rejection‐mediated cellular inactivation, tumorigenicity, and disease transmission.

**FIGURE 4:**
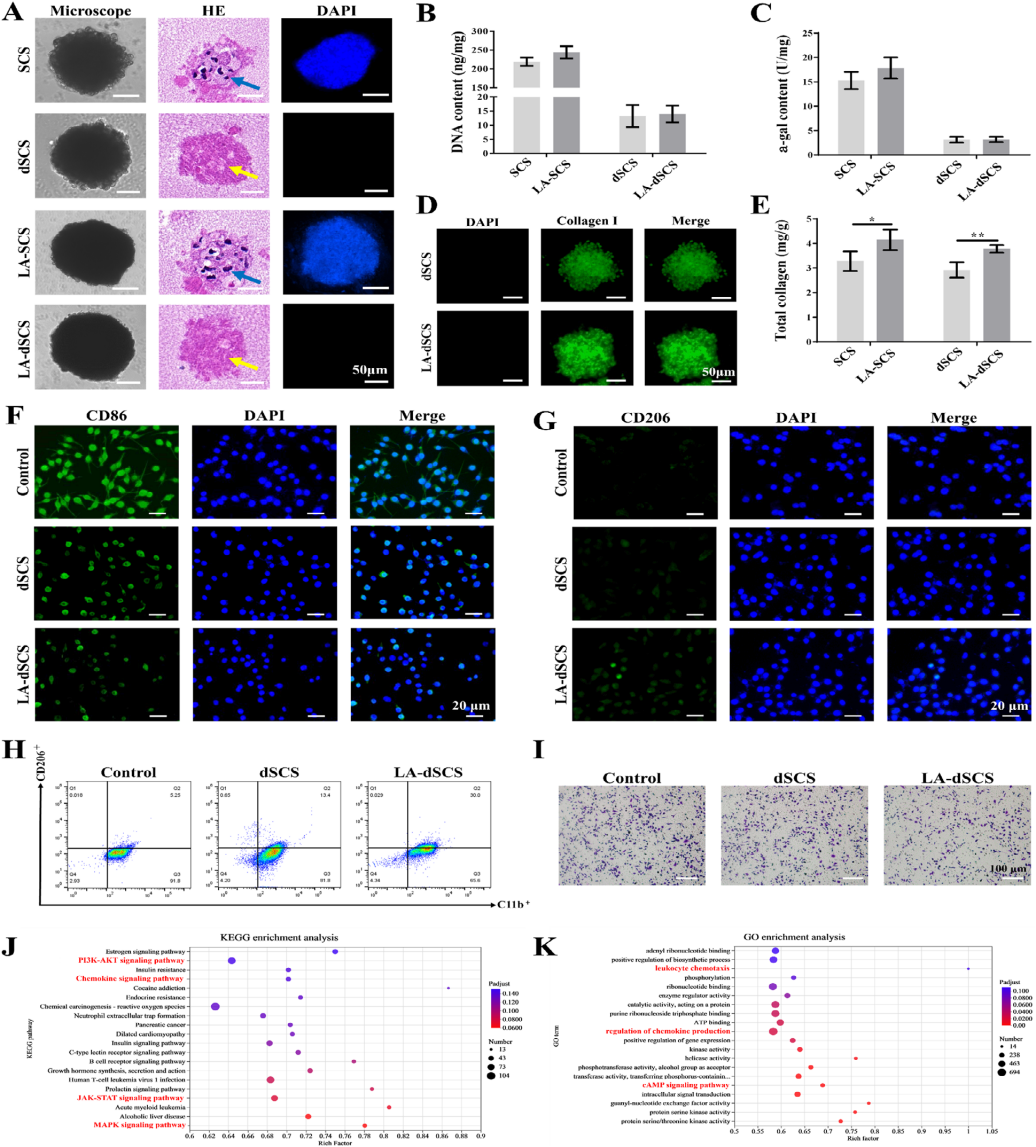
Fabrication and biological characterization of LA‐dSCS. (A) Gross morphology, HE, and DAPI staining confirmed near‐complete cell removal with preserved spheroid architecture. (B) Quantitative DNA content analysis. (C) Quantitative α‐gal content analysis. (D,E) COL I immunofluorescence staining and quantitative analysis demonstrated retained ECM integrity post‐decellularization. (F,G) CD86/CD206 immunofluorescence staining confirmed better immunomodulatory capacity of LA‐dSCS. (H) Flow cytometric analysis of CD206 expression in macrophages. (I) Transwell migration assay showed enhanced inhibition of macrophage migration by LA‐dSCS. (J,K) Proteomic analysis revealed LA‐dSCS modulated macrophage polarization, homeostasis, autophagy, and migration to achieve immunomodulation. One‐way analysis of variance (B,C,E) was used for statistical analysis; *n* = 5 for B, C, E, and H, *n* = 3 for J and K; All data were depicted as means ± SD; ^*^ *p* < 0.05, ^**^ *p* < 0.01.

Gross observation (Figure 4A) showed both groups maintained intact spherical structures post‐decellularization. HE and DAPI staining revealed significant nuclear elimination (blue arrows indicating nuclei, yellow arrows showing decellularized areas). Quantitative DNA and α‐gal assays confirmed a drastic reduction of immunogenic substances, indicating near‐complete removal of immune‐related substances (Figure 4B,C). Immunofluorescence and quantitative assays (Figure 4D,E) showed higher collagen content in LA‐SCS‐derived spheroids before/after decellularization, demonstrating stronger ECM biosynthesis. The decellularization protocol preserved bioactive substances without substantial loss.

#### 2.2.2 Immunomodulatory Function Assays

After successful isolation and culture of M1 macrophages (Figures S10–S13), CD86 immunofluorescence (Figure 4F; Figure S14) showed reduced pro‐inflammatory signals in LA‐dSCS‐treated M1 macrophages, while CD206 staining (Figure 4G; Figure S14) indicated enhanced M2 polarization. Flow cytometry confirmed higher CD206 expression in LA‐dSCS group (Figure 4H), demonstrating stronger M2 polarization capacity. LA‐dSCS significantly reduced macrophage migration compared to dSCS and Control groups (Figure 4I), likely via downregulating migration‐related receptors and cytokines, consistent with decreased M1 polarization and chemokine secretion.

The aforementioned results have demonstrated the stronger immunomodulatory capacity of the LA‐dSCS group. To unravel the underlying mechanism, we performed proteomic analysis [49]. A total of 3599 differentially expressed proteins (2133 upregulated, 1466 downregulated) were identified, with high inter‐sample consistency (Figure S15). KEGG pathway enrichment analysis highlighted significant activation of immune‐regulatory networks, including PI3K‐AKT, JAK‐STAT, and MAPK signaling pathways (Figure 4J).

PI3K‐AKT Pathway: Central to cell growth and metabolic regulation, PI3K phosphorylates PIP2 to PIP3, recruiting Akt to phosphorylate downstream targets. Class III PI3K forms tetrameric Complexes I and II: Complex I drives autophagosome biogenesis, while Complex II enhances autophagosome‐lysosome fusion via endosomal maturation, modulating macrophage autophagy and phagocytosis [50, 51].

MAPK Pathway: As a conserved serine/threonine kinase family, MAPK regulated pro‐inflammatory cytokines (TNF‐α, IL‐1, IL‐6) critical for macrophage polarization. It also modulated chemokine/adhesion molecule expression, influencing immune cell activation and migration [52].

JAK‐STAT Pathway: Essential for immune homeostasis, JAK‐STAT maintained cellular states via epigenetic/transcriptional regulation and rapid immune responses [53]. LA‐dSCS likely transmitted chemical signals to macrophage nuclei through JAK‐STAT, activating genes that regulated intracellular protein interactions and basal functions (immunity, proliferation, apoptosis), promoting homeostasis.

Functional annotation revealed differentially expressed proteins in signal transduction, transport, and catabolic metabolism. Enrichment of cell chemotaxis‐related genes aligned with Transwell migration data, indicating altered chemotactic capacity.

GO analysis identified macrophage‐relevant terms: leukocyte chemotaxis, biosynthetic process regulation, and kinase activity (Figure 4K). Leukocyte chemotaxis enrichment suggested regulated migration protein expression in LA‐dSCS‐treated macrophages, while biosynthetic/kinase activity terms indicated differential regulation of intracellular synthesis and phosphorylation, impacting protein function. GO annotation further showed comprehensive regulation of cellular processes, metabolism, and transcription (Figure S16). Collectively, LA‐dSCS bioactive substances modulated intracellular signaling and protein networks, altering macrophage polarization, homeostasis, autophagy, and migration. This mechanistic insight underscores LA‐dSCS potential for diabetic tissue defect repair.

To validate the findings of proteomic analysis, we performed western blot and ELISA for inflammation‐ and migration‐related proteins. As shown in Figure S17, protein expression differed significantly in the following aspects: (1) NLRP3 inflammasome activation was inhibited, reducing the inflammatory response [54]; (2) The current experimental results revealed upregulated expression of Nrf2 and TGF‐β1 and downregulated expression of IL‐1β. Nrf2, a key antioxidant transcription factor, regulated antioxidant enzyme genes to enhance cellular antioxidant capacity [55], promoting macrophage polarization toward an anti‐inflammatory phenotype; (3) MAPK signaling pathway conduction was modulated, interfering with pro‐inflammatory signaling in M1 macrophages and decreasing inflammatory factor production; (4) IL‐8 and CCL2 expression was lower in the LA‐dSCS group (consistent with Transwell results), reducing macrophage migration and preventing excessive accumulation. ELISA results in Figure S18 were consistent with western blot findings.

### 2.3 Fabrication and Characterization of LA‐dSCS Granular Gel

#### 2.3.1 Synthesis and Analysis of LA‐Grafted HA (HALA)

To confirm successful HALA synthesis, FTIR spectroscopy was performed. As shown in Figure S19A, a characteristic carbonyl stretching vibration band about 1700 cm^−^^1^ (ester group) and distinct C‐O‐C stretching of LA ester was observed, indicating successful grafting of LA onto HA. HALA retained HA‐specific peaks, demonstrating that grafting preserved the native HA architecture, consistent with successful conjugation. In addition, the ^1^H NMR results further confirmed the successful synthesis of HALA, with a modification rate of 84.6% (Figure S19B). In aqueous solution, HALA presented as a clear, flowable liquid (Figure S19C). Upon UV irradiation (365 nm), it rapidly crosslinked into a non‐flowing hydrogel that maintained shape when tilted, confirming excellent photocurability without additional or extra photosensitive small molecules.

#### 2.3.2 Synthesis and Analysis of LA‐dSCS Granular Gel

**FIGURE 5:**
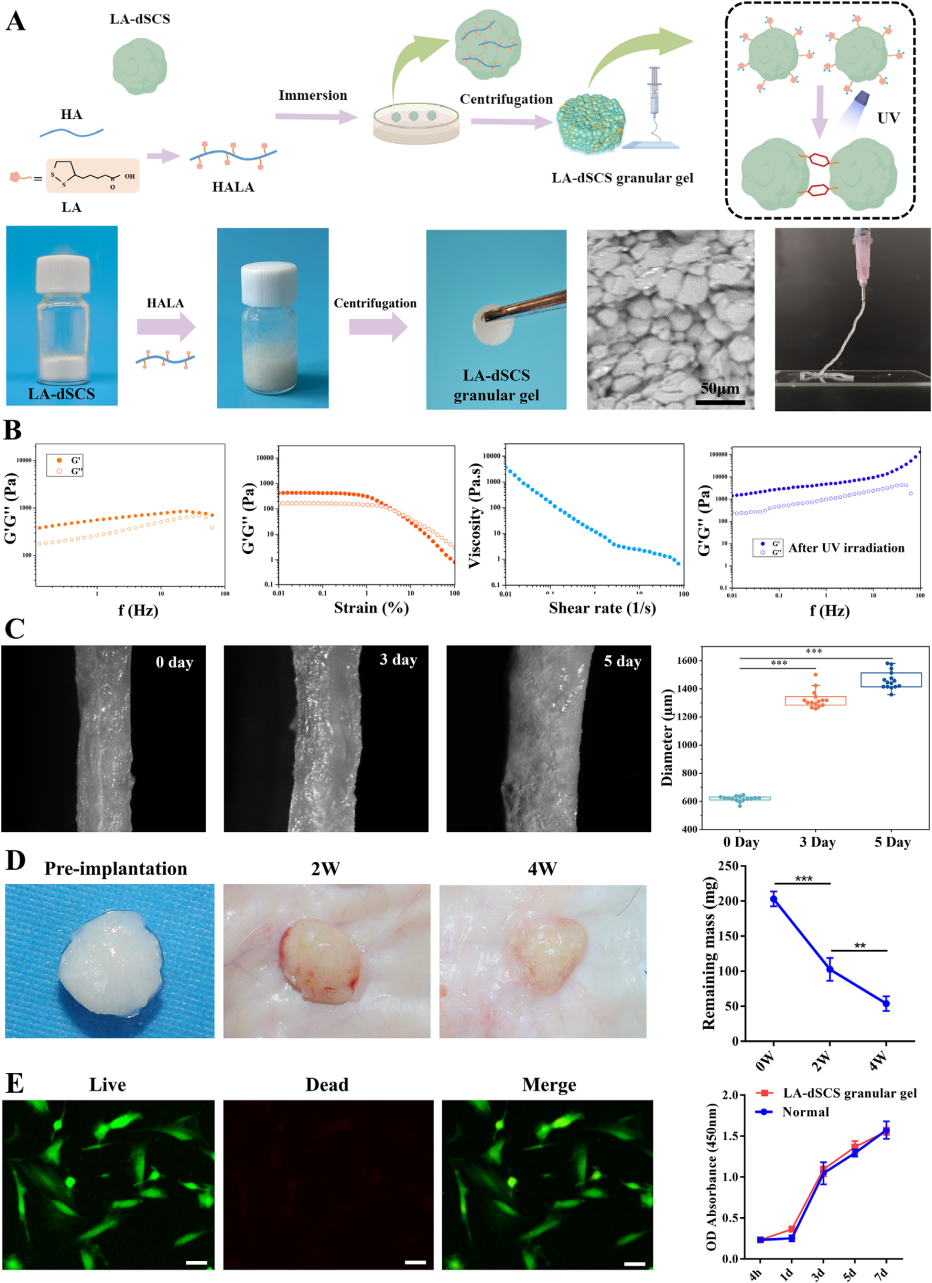
Fabrication and characterization of LA‐dSCS granular gel. (A) The construction process of the LA‐dSCS granular gel. (B) Rheological characterization (frequency, strain, and share rate tests). (C) Stereomicroscope photographs and diameter assessment of the extruded LA‐dSCS granular gel after immersion in PBS. (D) In vivo degradation evaluation (gross morphology and quantitative mass loss). (E) Live/dead staining and CCK‐8 assay for cytotoxicity evaluation. One‐way analysis of variance (C,D) was used for statistical analysis; *n* = 5 for D and E; All data were depicted as means ± SD. ^**^ *p* < 0.01, ^***^ *p* < 0.001.

Then we mixed LA‐dSCS powder with HALA solution to allow the powder to absorb HALA molecules, followed by centrifugation to yield a bulk granular gel with dense packing of particles (Figure 5A). The assessment results of injectability demonstrated that it exhibited thixotropic properties, which enabled a “solid‐to‐liquid” transition under shear force, thus facilitating extrusion and injection (Figure 5A).

Rheological analysis further confirmed the characteristics of the granular gel. As shown in Figure 5B, G' being greater than G'' indicated that the granular gel formed by particle aggregation was a typical bulk hydrogel. Under the action of shear force, G' becoming less than G'' means that the granular gel has the fluidity of a liquid (Figure 5B). At the same time, as the shear rate increases, the granular gel exhibited a significant shear thinning characteristic (Figure 5B). After UV irradiation, G' significantly increases compared to before irradiation, indicating that HALA undergoes cross‐linking, thereby enabling the granular gel to form a stable filling at the defect site (Figure 5B).

After immersion in PBS, the granular gel swelled while maintaining structural integrity at 3 days and 5 days (Figure 5C), confirming post‐injection stability. Then, the swelling ratio increased progressively, exhibiting a rapid initial phase (144.4 ± 12.1% at 24 h) followed by slower expansion (170.0 ± 8.1% at 48 h) and equilibrium at 96 h (207.4 ± 12.1%) (Figure S20), indicating saturation of the LA‐dSCS granular gel network.

Subcutaneous implantation in diabetes rats showed degraded from white to light yellow, with mass loss to 50% (102.6 ± 8.14 mg) at 2 weeks and 26.9% (53.75 ± 5.23 mg) at 4 weeks (Figure 5D). No severe immune rejection or tissue lesions were observed. Histopathological analysis of liver, heart, spleen, lung, and kidney at 4 weeks (Figure S21) revealed no significant alterations, confirming biosafety. Live/dead staining (Figure 5E) showed minimal cell death in granular gel extract, while CCK‐8 assay (Figure 5E) demonstrated time‐dependent cell proliferation, indicating favorable biocompatibility and lack of cytotoxicity.

### 2.4 Evaluation of Tissue Defect Repair Efficacy in Diabetic Rats

#### 2.4.1 Wound Healing Assessment

**FIGURE 6:**
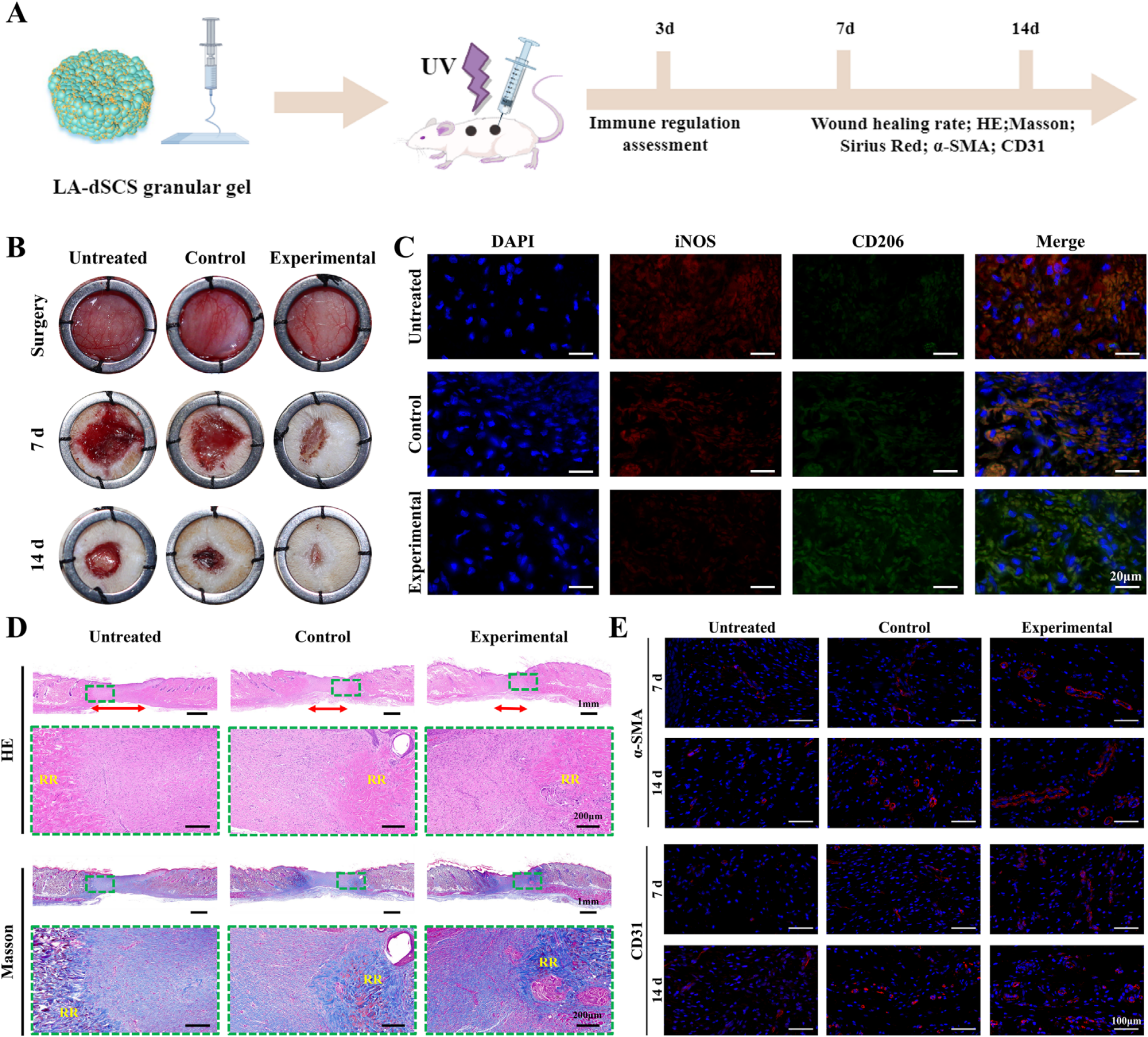
Diabetic wound repair efficacy evaluation. (A) Schematic illustration of wound repair assessment strategy. (B) Gross morphology analysis demonstrated the fastest wound closure in the Experimental group. (C) Immunofluorescence staining for iNOS and CD206, reflecting macrophage polarization states. (D) HE and Masson trichrome staining at day 14, showing better healing kinetics and collagen deposition in the Experimental group. (E) α‐SMA and CD31 immunofluorescence results, indicating enhanced myofibroblast activation and angiogenesis in the Experimental group. *RR: Regeneration Region*.

As shown in Figure 6B and Figure S22, the Untreated group exhibited extensive unhealed areas with copious inflammatory exudate at day 7 post‐surgery, while the Control group showed reduced wound area but persistent exudate and poor healing. Notably, the Experimental group demonstrated accelerated healing with abundant neotissue formation and minimal exudate. By day 14, the Experimental group achieved near‐complete closure with normalized skin pigmentation and hair regrowth, in stark contrast to the suboptimal repair in the Control groups.

Immunostaining revealed abundant iNOS^+^ M1 macrophages and sparse CD206^+^ M2 macrophages in the Untreated group (Figure 6C). The Experimental group potently suppressed M1 polarization, as evidenced by peak IL‐4 expression (anti‐inflammatory) and minimal IL‐1β (pro‐inflammatory) (Figures S23 and S24), indicating skewed M2 polarization.

HE staining (Figure 6D; Figure S25) revealed improved repair in all groups by day 14 vs. day 7. The Experimental group exhibited fewer unhealed area and reduced inflammatory cell infiltration, reflecting accelerated tissue regeneration relative to controls. As a key ECM substance, collagen organization directly impacts wound healing quality. Masson trichrome staining (Figure 6D; Figure S25) showed sparse, disorganized collagen fibers in the Untreated group, modest fiber increase but loose alignment in the Control group, and dense, orderly collagen deposition in the Experimental group. α‐SMA immunofluorescence demonstrated robust myofibroblast activation in the Experimental group, driving wound contraction at days 7 and 14 (Figure 6E). CD31 staining revealed significantly higher vascular density in the Experimental group, supplying essential nutrients and oxygen to support healing (Figure 6E).

#### 2.4.2 Skull Defect Model Establishment

As shown in Figure S26A, following successful rat anesthesia, the scalp incision exposed the calvarial surface. Using an electric drill, we created a standardized skull defect (Figure S26B), followed by injection and UV photocuring of the LA‐dSCS granular gel. (Figure S26C).

**FIGURE 7:**
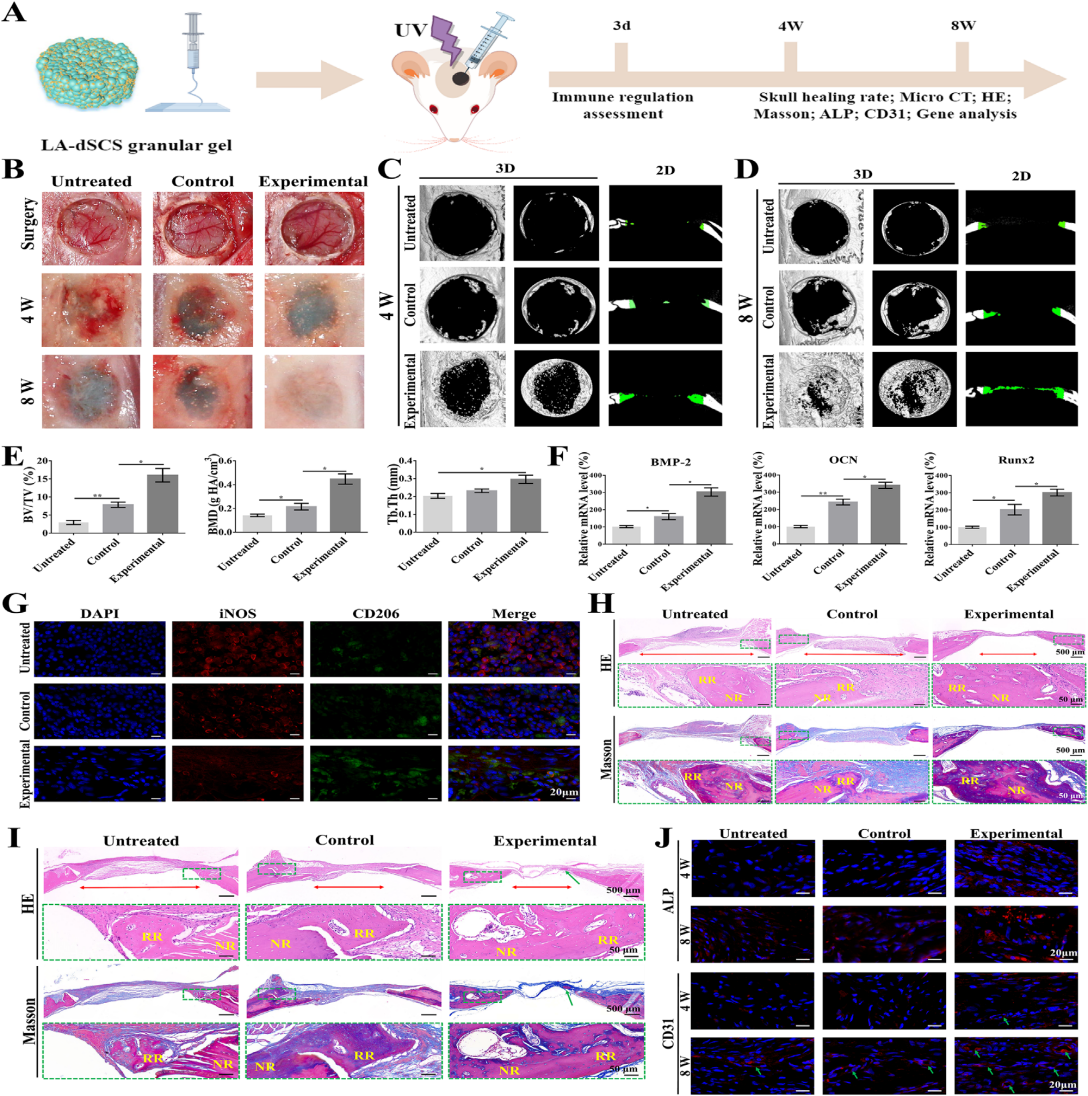
Evaluation of diabetic skull defect repair. (A) Schematic illustration of the skull repair procedure. (B) Gross morphology analysis demonstrated the fastest defect closure in the Experimental group. (C–E) Micro‐CT assessments showing better BV/TV and BMD in the Experimental group at 4 and 8 weeks. (F) The assessment of gene expression indicated that the Experimental group has the highest expression of genes related to bone regeneration. (G) iNOS/CD206 immunofluorescence staining confirmed enhanced immunomodulatory capacity, with skewed M2 macrophage polarization in the Experimental group. (H,I) HE and Masson trichrome staining at 4 and 8 weeks, respectively, showing accelerated bone trabecular formation, dense collagen deposition, and improved tissue integration in the Experimental group. (J) ALP/CD31 immunofluorescence assessment. *NR: Native region; RR: Regenerated region*. One‐way analysis of variance (E,F) was used for statistical analysis; *n* = 5; All data were depicted as means ± SD; ^*^ *p* < 0.05, ^**^ *p* < 0.01.

At 4‐ and 8‐weeks post‐surgery, we evaluated euthanized rats. At 4 weeks (Figure 7B), the Untreated group showed extensive unhealed defects with disordered fibrotic tissue due to diabetic pathological microenvironment. The Control group exhibited partial bone repair with a semi‐transparent central area, while the Experimental group showed significantly larger bone regeneration. By 8 weeks, the Untreated and Control groups still had substantial defects, whereas the Experimental group displayed the best repair, with neotissue color matching adjacent bone.

To further characterize the repair efficacy, we performed Micro‐CT for 3D reconstruction and 2D cross‐sectional analysis (green regions denote the repaired areas). At 4 weeks post‐surgery, Micro‐CT analysis showed that the Untreated group exhibited large lucent defects with minimal new bone ingrowth, while the Control group had reduced cavity volume with sparse, patchy bone formation. In contrast, the Experimental group demonstrated dense bone filling throughout the defect, including scattered trabeculae in the central region. At 8 weeks (Figure 7D), the Experimental group showed the most defects filled with new bone, though not fully normalized. Quantitative analysis (Figure 7E) showed significantly higher BV/TV and BMD in the Experimental group. Tb. Th trended highest in the Experimental group, though not statistically distinct from the Control.

For immunomodulatory evaluation of skull repair, the Experimental group demonstrated optimal immune regulation (Figure 7G), with LA‐dSCS potently inhibiting pro‐inflammatory macrophages. TNF‐α and TGF‐β1 immunofluorescence (Figures S27 and S28) confirmed better anti‐inflammatory effects.

HE and Masson trichrome staining (Figure 7H,I) at 4 and 8 weeks revealed that the Untreated group showed dense inflammatory infiltration and fibrous tissue filling at 4 weeks, with persistent unhealed defects and sparse collagen at 8 weeks. The Control group exhibited reduced inflammation and minimal bone formation at 4 weeks, with improved but suboptimal bone growth and collagen alignment at 8 weeks. In contrast, the Experimental group demonstrated attenuated inflammation, abundant trabecular bone, and orderly collagen deposition at 4 weeks, progressing to significantly regressed defects, natural tissue integration, and dense ECM remodeling (evident by blue‐stained collagen fibers) at 8 weeks, with central osteogenesis marked by green arrows. Regarding the integration of neotissue with adjacent native region, neither the Control nor Experimental group showed satisfactory integration at 4 weeks. By 8 weeks, the Control group exhibited no notable improvement, whereas the Experimental group demonstrated significantly enhanced tissue integration with adjacent native region. To assess osteoblast activity and angiogenic potential, ALP and CD31 immunofluorescence staining were performed on samples at 4 and 8 weeks post‐surgery. As shown in Figure 7J, the Experimental group exhibited markedly stronger ALP and CD31 fluorescence signals than Control groups, with widespread distribution in the defect area (green arrows). This indicated that bioactive substances in LA‐dSCS remodeled the local microenvironment, enhancing osteoblast function and neovascularization to accelerate bone matrix synthesis and promote rapid defect closure [56].

Following implantation of LA‐dSCS granular gel into diabetic tissue defects, the Experimental group exhibited optimal wound and cranial bone defect repair, outperforming controls in closure rate, immunomodulation, angiogenesis, and collagen deposition. In general, this efficacy stems from: (1) LA‐dSCS multifunctionality as a bioactive construct with intact 3D architecture and ECM substances (collagen, laminin, growth factors) provided a permissive microenvironment for cell adhesion/differentiation; (2) LA‐dSCS granular gel's biological safety, which prolongs active factor retention upon LA‐dSCS conjugation, enabling sustained immunomodulation, collagen deposition, and angiogenesis.

For cranial bone defects, although complete defect repair has not been achieved, it still exhibits significant microenvironmental regulatory effects, better angiogenesis and osteogenic capabilities, thereby achieving a better promoting effect on repair.

However, as an early‐stage proof‑of‑concept study, the current research still has the following unresolved limitations: (1) The more in‐depth mechanistic exploration of LA‐mediated cell function reconstitution, including mitochondrial metabolism and ferroptosis regulation need to be further evaluated; (2) The capacity of the granular gel production was relatively small, and the steps were refined. Further optimization is still needed in the future in terms of standardization and scale‐up. In addition, the degradation rate of the LA‐dSCS granular gel at the defect site needs to be further evaluated; (3) The absence of a control group using the “gold standard” material represents a limitation of the current research. Among them, autologous bone grafting remains the “gold standard”, while commercial decalcified bone matrix (DBM) scaffolds (such as OsteoSelect) or β‐tricalcium phosphate (β‐TCP) scaffolds were also applied [57, 58, 59, 60]. Autologous grafting has inherent drawbacks such as donor site injury, limited sources of bone harvesting for large‐area defects, and difficulty in matching the skull geometry [58]. Commercial scaffolds were often restricted by issues like infection, graft migration, and poor bone repair outcomes [61]. In subsequent studies, direct comparisons with other restoration methods can further clarify its potential for clinical translation; (4) In future studies, more biological replicates (further enhance the reliability of the data.), longer repair observation periods (evaluate the long‐term bone stability of the biological materials, the late mineralization process, and the final tissue integration effect), and large animal (simulate the physiological microenvironment and tissue repair characteristics of humans) experiments will be required in animal studies.

## 3 Conclusion

With the aim of developing a novel tissue engineering biomaterial capable of exerting multiple regulatory effects on the microenvironment of diabetic tissue defects, we successfully constructed a novel 3D biomimetic engineered bioactive substance aggregate (LA‐intervened decellularized stem cell spheroids, LA‐dSCS). It exhibited a higher content of bioactive substances and better immunomodulatory potency, and was capable of activating signaling pathways, including macrophage autophagy. Then, we have further achieved the successful preparation of the LA‐grafted HA(HALA) photosensitive unit. Following its pre‐assembly with LA‐dSCS, dense packing occurs, resulting in the formation of a LA‐dSCS granular gel. The in vivo results demonstrated superior repair effects on both cutaneous wounds and cranial bone defects. While future studies were warranted to address clinical translation challenges, the current results provided proof‑of‑concept for targeted wound/skull repair of rat.

## 4 Experimental Sections

### 4.1 Experimental Method

Biocompatibility assessment of LA and LA co‐culture: We isolated and expanded ADSCs to passage 2 (P2) as described previously, using a laboratory‐formulated stem cell medium [31]. All animal procedures were approved by the Institutional Ethics Committee (No. 2025‐DW‐SB‐086). For cytotoxicity evaluation, we plated ADSCs suspensions onto glass coverslips and allocated to two groups: LA (‐) group and LA (+) (50 µm, Sinopharm, Beijing, China) group. Cytotoxicity assay (CCK‐8, C0038, Beyotime) and live/dead staining with Calcein‐AM/PI kit (CA1630, Solarbio) at 1‐, 2‐, and 3‐days post‐seeding. Subsequently, HE staining for cell morphology and distribution, phalloidin‐FITC labeling for Filamentous‐actin, and TUNEL assay to assess cellular morphology, cytoskeletal architecture, and apoptosis, respectively.

To assess stemness maintenance and anti‐inflammatory capacity, LA‐pretreated ADSCs were exposed to an in vitro diabetic microenvironment (25 mm glucose + 1 mm H_2_O_2_), designated as LA‐intervened ADSC group (LA‐ADSC group); non‐LA treated cells served as ADSC group. After 12 h, we performed immunofluorescence staining and quantitative real‐time PCR (qRT‐PCR) to evaluate stemness markers (NANOG, OCT4, SOX2) and pro‐inflammatory cytokine TNF‐α. Primer sequences were detailed in Table S1.

Cell spheroid fabrication and characterization: we seeded LA‐co‐cultured ADSCs (3000 cells/well) onto ultralow attachment plates (3473, Corning, NY, USA) for 10‐day spheroid formation, yielding LA‐SCS group; non‐induced cells formed stem cell spheroid group (SCS group). We used microscope and live/dead viability staining on days 3, 5, 7, 10 to assess the gross appearance and growth status of the spheroids. Following 10‐day culture, we performed collagen type I (COL I) immunofluorescence, paracrine gene expression, and ELISA analysis (bFGF, HGF, IGF‐1), and RNA‐seq transcriptomic profiling for spheroids. For RNA‐seq analysis, we extracted the total RNA from the cell spheroids using TRIzol Reagent (Invitrogen, Carlsbad, CA, USA), after in vitro culture for 10 days. The TruSeq RNA Sample Preparation Kit (Illumina, San Diego, CA, USA) was used to establish the library. The Illumina HiSeq XTEN/NovaSeq 6000 sequencing platform was then used for high‐throughput sequencing of the library. To identify the differentially expressed genes (DEGs), we used transcripts per million reads (TPM) to calculate the expression level of each transcript. Finally, Kyoto Encyclopedia of Genes and Genomes (KEGG) pathway analysis and Gene Ontology (GO) functional enrichment were carried out by Goatools and KOBAS. Subsequently, we performed Western bolt analysis to further confirm the results of RNA‐seq on several important signaling pathways (Wnt, AGE‐RAGE, and MAPK signal pathway).

Following 10‐day in vitro culture, we subjected LA‐SCS and SCS groups to a simulated diabetic microenvironment (high glucose + oxidative stress) for live/dead cell staining. We performed flow cytometric apoptosis analysis on both groups, with a Normal group (spheroids cultured in standard medium) included as a reference. Immunofluorescence staining for TNF‐α/IL‐4 and western blot analysis were conducted to characterize inflammatory responses. We quantified reactive oxygen species (ROS) levels using flow cytometry to evaluate oxidative stress status. To assess apoptosis, hypoxia adaptability, and angiogenic potential, we used qRT‐PCR to evaluate mRNA expression of BAX (apoptosis), HIF1‐α (hypoxia), and VEGF (angiogenesis) in spheroids [62]. Primer sequences were provided in Table S1.

Decellularization evaluation: Following 10‐day in vitro culture, we used PBS to wash spheroids and then decellularization. Then, we collected spheroids in centrifuge tubes, resuspended in PBS, and frozen at −80°C overnight. Thawing was performed in a 37°C water bath, with three freeze‐thaw cycles repeated. Spheroids were then incubated in 1% Triton X‐100 (v/v) PBS for 30 min, followed by three PBS washes. Decellularized SCS was designated as dSCS group, and decellularized LA‐SCS as LA‐dSCS group.

Macroscopic morphological observation under microscopy assessed structural integrity. Agarose‐embedded spheroids were subjected to HE and DAPI staining to evaluate nuclear elimination. COL I immunofluorescence staining compared collagen preservation before/after decellularization. PicoGreen assay (P7589, Invitrogen, Carlsbad, USA) compared DNA content to confirm DNA removal. α‐galactosidase (α‐gal) content, a marker of immunogenicity, was measured by ELISA (70101, Sanyao Science, China). We quantified total collagen via alkaline hydrolysis (A030‐2‐1, Jiancheng Bio, Nanjing, China).

Immunomodulatory function assays: we isolated primary macrophages from mouse peritoneum and characterized by flow cytometry for purity [63]. Then, we induced M1 polarization with 100 ng/mL LPS + 50 ng/mL IFN‐γ for 12 h. Immunofluorescence (CD86, CD206) and gene expression (iNOS, TNF‐α, IL‐1β) via qRT‐PCR (primer sequences in Table S1) validated induction efficiency.

M1 macrophages were co‐cultured with dSCS or LA‐dSCS for 48 h in the following groups: Control group (no treatment), dSCS group (co‐culture with dSCS), and LA‐dSCS group (co‐culture with LA‐dSCS). We analyzed CD86/CD206 expression by immunofluorescence and flow cytometry. Migration ability was evaluated via Transwell assay: upper chambers contained FBS‐free medium with dSCS/LA‐dSCS extracts, and lower chambers contained 0.6 mL complete medium, cultured for 48 h.

To elucidate the mechanistic basis for the distinct immunomodulatory profiles, we performed proteomic analyses on macrophages following intervention with dSCS or LA‐dSCS. Differentially expressed proteins were annotated using GO and KEGG databases, with significant enrichment defined as corrected *P* < 0.05. NLRP3, Nrf2, MAPK, TGF‐β1, IL‐1β, IL‐8, and CCL2 were quantified by Western Blot to validate proteomic findings. To assess cytokine secretion profiles, we performed ELISA (TGF‐β, IL‐1β, IL‐8, CCL2) per the manufacturer's instructions.

Synthesis and analysis of LA‐grafted HA (HALA): First, 0.628 g LA was dissolved in 3 mL N, N‐dimethylformamide (DMF) at room temperature, followed by the addition of 0.485 g N, N′‐carbonyl diimidazole (CDI). The mixture was stirred at 25°C for 1 h to activate carboxyl groups of LA, yielding lipoyl imidazole (LA‐IM). Then, HA was dissolved in formamide at 95°C, cooled to room temperature, and mixed with 4‐dimethylaminopyridine (DMAP) and LA‐IM. After stirring at 25°C for 4 h, the crude product was neutralized with potassium dihydrogen phosphate solution, dialyzed to remove solvents/impurities, and lyophilized at −25°C under light protection to obtain HALA.

To confirm HALA synthesis, we performed Fourier transform infrared (FTIR) analysis. Freeze‐dried HALA (−80°C, 24 h) was ground into powder, mixed with 10 mg KBr, and pressed into pellets. FTIR spectra were acquired using a Nicolet iS10 spectrometer (Thermo Fisher Scientific, Germany) in transmission mode (400–4000 cm^−^^1^, 4 cm^−^^1^ resolution, 16 scans). To further confirm HALA synthesis, we performed Proton nuclear magnetic resonance spectroscopy (^1^H NMR). 500 µL of deuterated water (D_2_O) dissolved a HALA sample (5 mg), with tetramethylsilane (TMS) serving as the internal standard. Qualitative and quantitative analyses of the samples were conducted using a nuclear magnetic resonance spectrometer (Avance 500 MHz, Bruker, Switzerland) with 128 scans. Following the processing of the ^1^H NMR data, we computed the degree of substitution via the MESTRENOVA software.

To further evaluate the photosensitivity of HALA, we dissolved HALA (0.03 g) in 1 mL DI water, added to silver ion solution (0.1 mg/mL), and irradiated at 365 nm to assess photo curability.

Synthesis and analysis of LA‐dSCS granular gel: we immersed LA‐dSCS (100 mg) in HALA solution and centrifuged to obtain LA‐dSCS granular gel. The microstructure of the granular gel was observed by SEM. To test the extrudability and continuity of the granular gel, the prepared granular gel was transferred into a syringe cylinder, and extrusion tests were conducted using an 18 G needle.

To further evaluate the rheological characterization, we analyzed LA‐dSCS granular gel (12 mm diameter × 5 mm height) on DHR‐3 rheometer (TA Instruments, USA) at 25°C, with frequency scanning (0.01–100 Hz) under 0.1% strain to determine storage modulus (G′) and loss modulus (G″). Strain sweep tests were carried out at a fixed frequency of 1 Hz, with the strain varying from 0.01% to 1000%. To study the shear‐thinning property, we examined the viscosity change of the granular gels as the shear rate was steadily increased from 0 to 300 s^−^^1^. Strain steps alternating between 1% and 100% at 1 Hz were implemented, and the variations of G′ and G″ with time and strain were recorded, respectively.

To further assess the stability, the morphology of LA‐dSCS granular gel was observed by stereomicroscopy (SZX10, Olympus, Japan) immediately, 3 days, and 5 days post‐extrusion. Then, we immersed Cylindrical LA‐dSCS granular gel in PBS (37°C) to perform swelling kinetics, and the swelling ratio was calculated as (Wt/W_0_) × 100%, where W_0_ = initial weight and Wt = weight at 24, 48, 72, 96 h.

LA‐dSCS granular gel (200 mg) was implanted subcutaneously in diabetic rats to perform in vivo degradation and safety. We quantified the remaining weight at 2 and 4 weeks and conducted HE staining of major visceral tissues (heart, liver, spleen, lung, kidney) at 4 weeks post‐implantation. In addition, we cultured ADSCs (P2) in gel extracts for 48 h to access biocompatibility, with live/dead staining (Calcein‐AM/PI) and CCK‐8 assay (450 nm absorbance) performed at 4 h, 1, 3, 5, and 7 days. The groups included the Normal group (normal stem cell medium) and LA‐dSCS granular gel group (extract‐treated).

Wound healing assessment: Male SD rats (200 g) from Shanghai Jiao Tong University College of Agriculture Internship Co., Ltd. were acclimated under SPF conditions for 1 week, fed a high‐fat/high‐sugar diet, and injected intraperitoneally with STZ (C1013, Solarbio, Beijing, China). The injection protocol for STZ was 40 mg/kg, 5 consecutive days, dissolved in sodium citrate buffer (S8050, Solarbio, Beijing, China). We confirmed diabetes by random blood glucose ≥ 16.7 mmol/L and polyuria/polydipsia/polyphagia on days 3, 7, 14, and 21 after injection.

Under anesthesia, we created 1 cm‐diameter circular wounds on rat backs, fixed with iron rings, and rats were randomized into: Untreated group, Control group (HALA implantation), and Experimental group (LA‐dSCS granular gel implantation). A total of 45 rats were allocated into 3 groups (*n* = 5 per group), with dressings replaced at 3‐day intervals. At day 3, we performed iNOS/CD206 (macrophage polarization) and IL‐1β/IL‐4 (inflammation) stained. At days 7/14, we evaluated healing by gross observation, HE, Masson's trichrome, and α‐SMA/CD31 immunofluorescence.

Skull regeneration assessment: Under general anesthesia, we used a scalpel to make a 2‐cm midline incision on the rat calvaria. A 5‐mm‐diameter circular defect was created using a sterile trephine bur, with continuous irrigation of sterile saline to prevent frictional damage to adjacent tissues. The groups and sample sizes were identical to those in the cutaneous wound model.

At day 3, we evaluated iNOS/CD206 (macrophage polarization) and TNF‐α/TGF‐β1 (inflammation). At weeks 4/8, we used Micro‐CT (Scanco Medical, 70 kV, 114 µA, 2048 × 2048 resolution) to calculate bone volume fraction (BV/TV), bone mineral density (BMD), and trabecular thickness (Tb.Th). Histological analysis included HE, Masson's trichrome, and ALP/CD31 immunofluorescence. At week 8, BMP‐2, OCN, and Runx2 expression was quantified by qRT‐PCR (primers in Table S1).

### 4.2 Statistical Analysis

Statistical analyses were performed using GraphPad Prism 8 software. Data were presented as mean ± standard deviation (mean ± SD). For intergroup comparisons, we used an independent sample *t*‐test for two‐group comparisons and one‐way analysis of variance (ANOVA) for multiple‐group comparisons. Statistical significance was defined as *P* < 0.05. The RNA sequencing experiment and the proteomics experiment were conducted with *n* = 3, while the rest of the experiments were carried out with *n* = 5.

## Conflicts of Interest

The authors declare no conflicts of interest.

## Supporting information




**Supporting file**: advs75165‐sup‐0001‐SuppMat.docx

## Data Availability

The data that support the findings of this study are available from the corresponding author upon reasonable request.
