# Supplementary material for: Lipoic Acid‐Intervened Decellularized Stem Cell Spheroid‐Based Injectable Granular Gel for Diabetic Tissue Regeneration
Source: Adv Sci (Weinh). 2026 Apr 7;13(36):e21924. doi: 10.1002/advs.202521924 (PMC13317787; doi:10.1002/advs.202521924)
Supplement: Supplementary file 1 — Supporting file: advs75165‐sup‐0001‐SuppMat.docx [file ADVS-13-e21924-s001.docx]

Supporting Information

**Lipoic Acid-intervened Decellularized Stem Cell Spheroid-based Injectable Granular Gel For Diabetic Tissue Regeneration**

Tao Wang^1, 3, a^, Haowei Fang^2, a^, Lili Qi^1, 3, a^, SONG MYOUNGSEOP^1, 3^, Aawrish Khan ^1, 3^, Lunli Gong ^1, 3^, Guangdong Zhou ^4, *^, Kunxi Zhang^1, 2, *^, Haiyan Cui^1, 3, *^

^a^ These authors contributed equally to this work.

^*^Corresponding authors: Haiyan Cui, Email: u2beauty1@sina.com; Kunxi Zhang, Email: zhangkunxi@shu.edu.cn; Guangdong Zhou, Email: guangdongzhou@126.com.

^1^ Department of Plastic and Cosmetic Surgery, Tongji Hospital, School of Medicine Tongji University, Shanghai 200092, P. R. China.

^2^ Department of Polymer Materials, School of Materials Science and Engineering, Shanghai University, Shanghai 200444, P. R. China.

^3^ Institute of Aesthetic Plastic Surgery and Medicine, School of Medicine, Tongji University, Shanghai, 200065, P. R China.

^4^ Department of Plastic and Reconstructive Surgery, Shanghai Ninth People’s Hospital, Shanghai Key Laboratory of Tissue Engineering, Shanghai Jiao Tong University School of Medicine, Shanghai 200011, P.R. China.


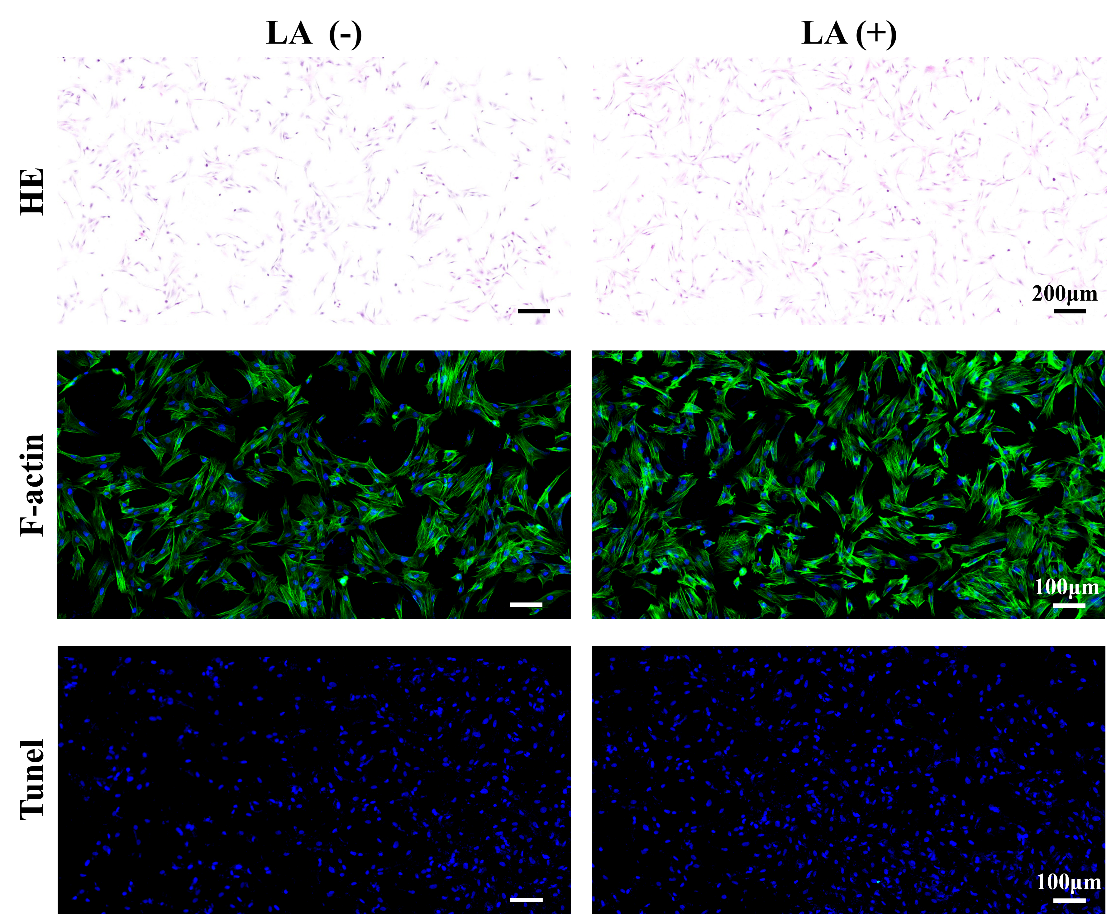


**Figure S1. Evaluation of cell slide staining following LA preprogramming.** HE staining revealed no significant abnormalities in cell morphology, with all cells exhibited a typical spindle shape and increased cellular density in the LA (+) group. F-actin staining demonstrated that the cytoskeleton in the LA (+) group was more intricately interwoven, accompanied by more robust actin synthesis and secretion. TUNEL staining indicated that LA exerted no significant effect on the apoptotic process of ADSCs, and the balance of cellular apoptosis was maintained.


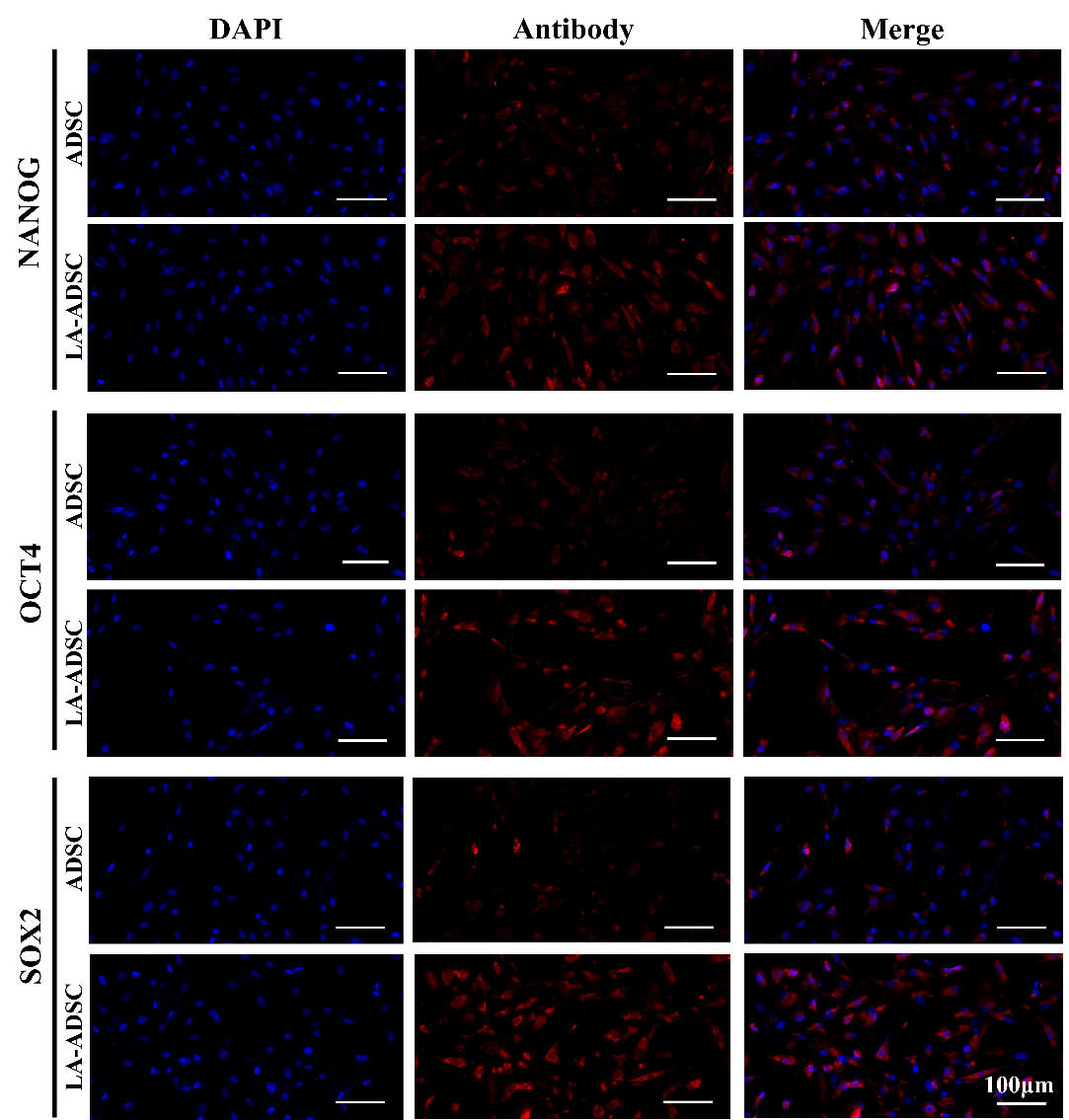


**Figure S2. Evaluation of stemness-related immunofluorescence staining of cells in a diabetic microenvironment.** The Experimental results showed that the fluorescence expression levels of NANOG, OCT4, and SOX2 in the LA-ADSC group were significantly higher than those in the ADSC group, with a corresponding increase in the proportion of positive cells. These findings indicated that ADSCs following LA intervention exhibited enhanced antioxidant capacity.


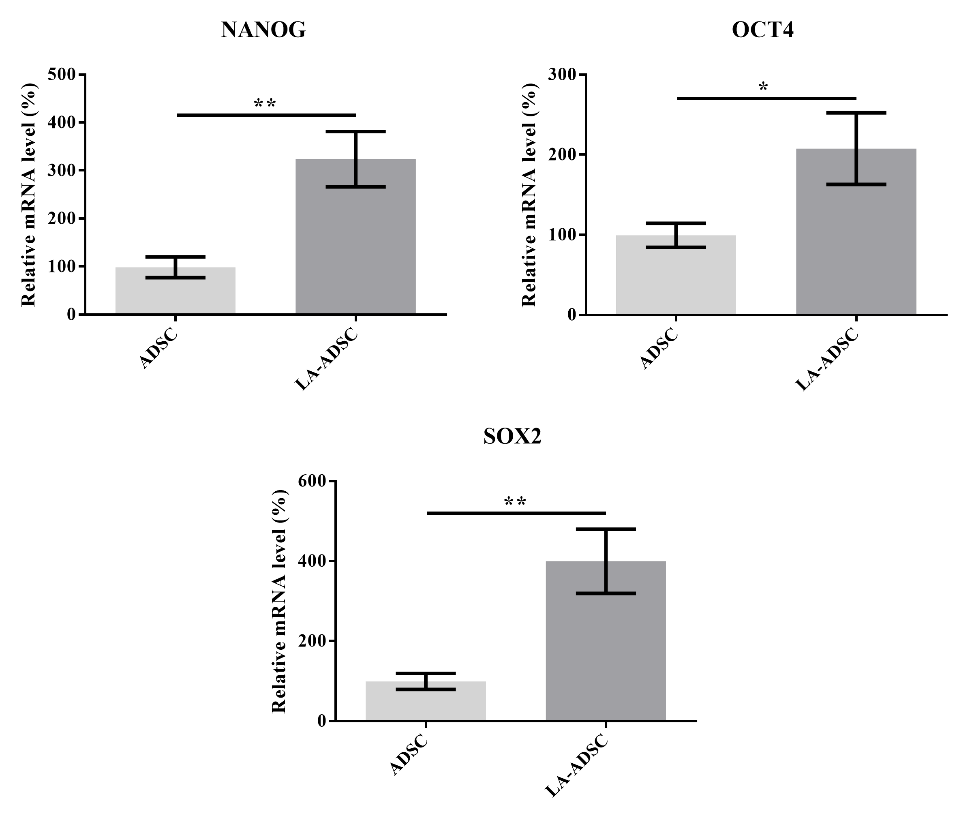


**Figure S3. Evaluation of stemness-related gene expression under a diabetic microenvironment.** Compared with the ADSC group, the LA-ADSC group showed significantly higher relative expression levels of NANOG, OCT4, and SOX2 genes, which was consistent with the results of immunofluorescence staining. Independent sample t test was used for statistical analysis; n = 5; All data were depicted as means ± SD; ^*^*p* < 0.05, ^**^*p* < 0.01.


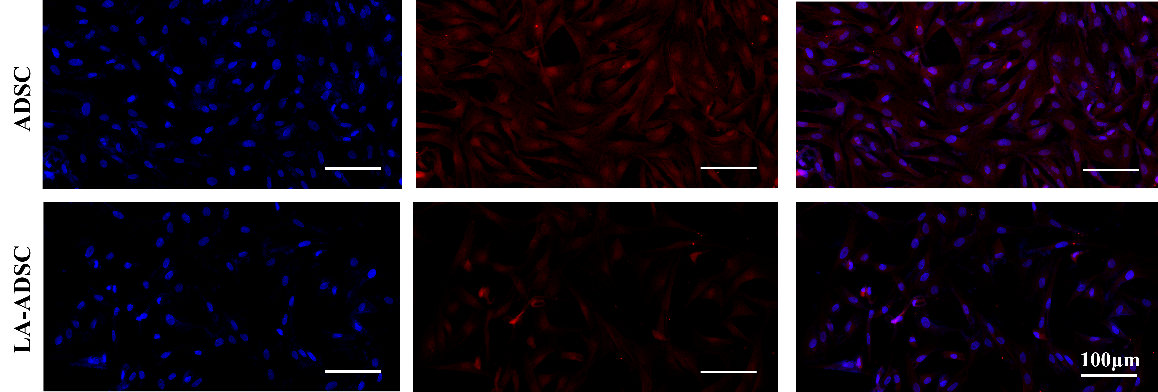


**Figure S4. Evaluation of TNF-α immunofluorescence staining in cells under a diabetic microenvironment.** The LA-ADSC group exhibited weaker fluorescence signal intensity corresponding to TNF-α protein, which further confirmed that LA preprogramming enhanced the anti-inflammatory capacity of the cells.


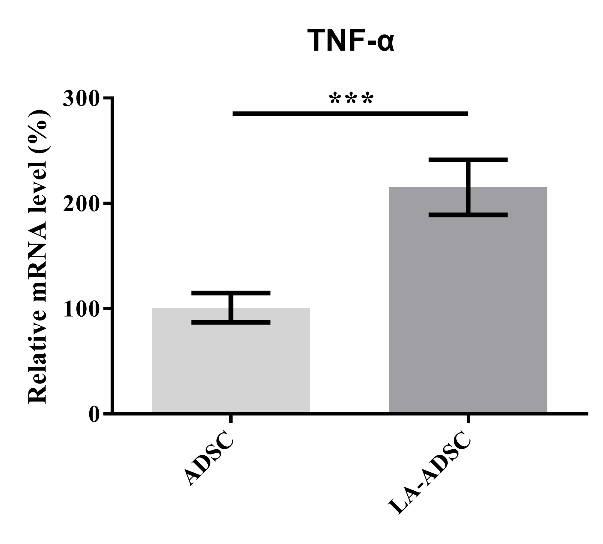


**Figure S5. Evaluation of TNF-α gene expression in cells under a diabetic microenvironment.** The relative expression level of the TNF-α gene in the LA-ADSC group was lower, which was consistent with the expression pattern observed in immunofluorescence staining. Independent sample t test was used for statistical analysis; n=5; All data were depicted as means ± SD; ^***^*p* < 0.001.


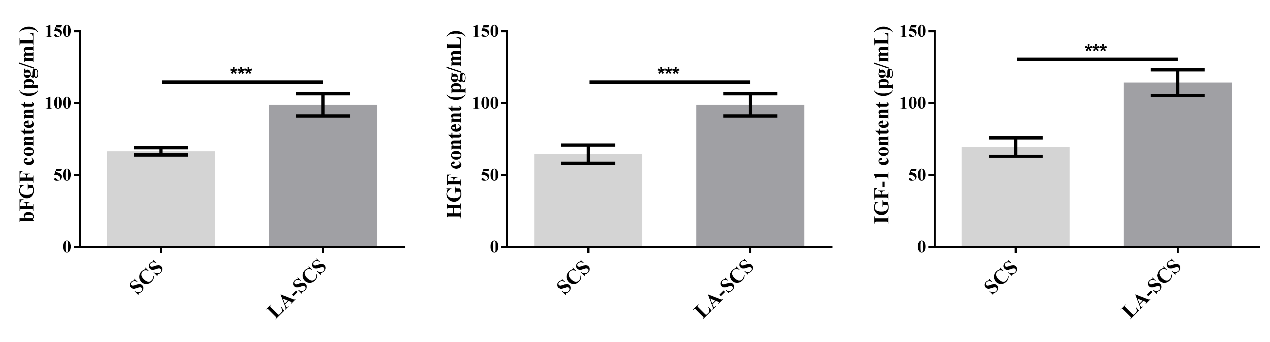


**Figure S6. ELISA assay of paracrine ability.** The results indicated that the LA-SCS group has a higher secretion capacity of bFGF, HGF, and IGF-1. Independent sample t test was used for statistical analysis; n = 5; All data were depicted as means ± SD; ^***^*p* < 0.001.


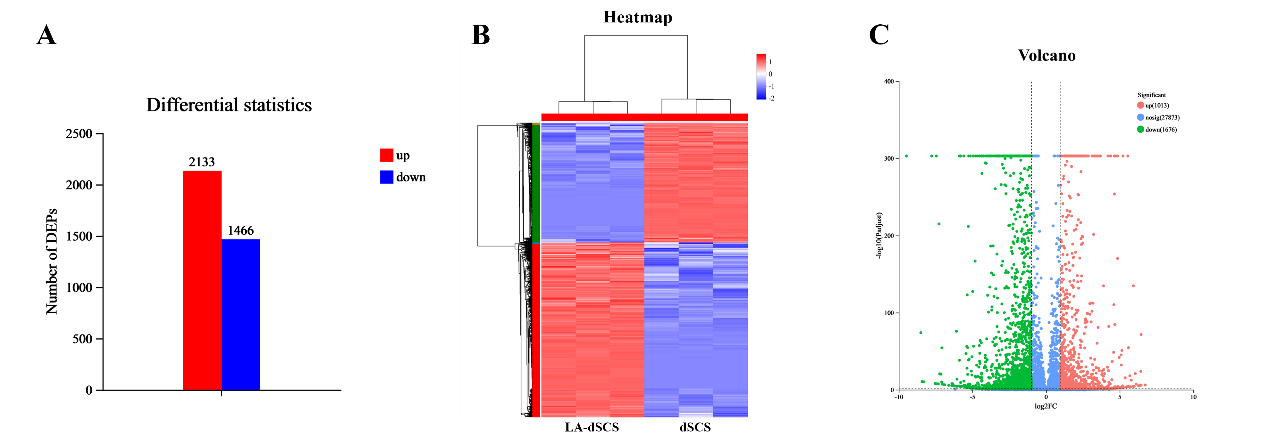


**Figure S7. Differentially expressed genes, heatmap and volcano plot analysis of RNA-seq data of LA-SCS and SCS groups.** (A) The results revealed a total of 2,689 differentially expressed genes, among which 1,013 genes were significantly upregulated and 1,676 genes were significantly downregulated. (B) The results demonstrated that samples in both groups exhibited good reproducibility, while distinct differences in gene expression were observed between groups. (C) The results indicated a substantial number of genes with differential expression. In the plot, each dot represented a specific gene, where those on the left correspond to downregulated genes and those on the right represented upregulated genes. n = 3.


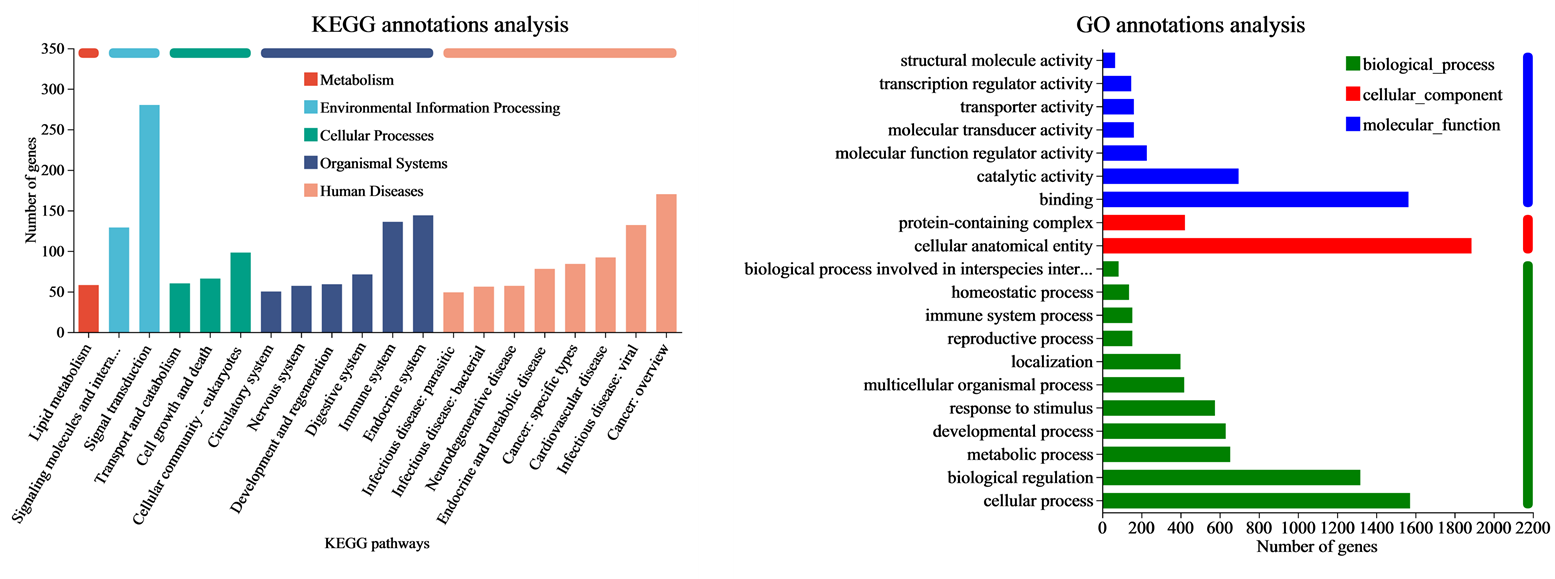


**Figure S8. KEGG and GO functional annotation analyses.** Functional annotation analyses revealed that differentially expressed genes were widely distributed across multiple categories, including metabolism, environmental information processing, cellular metabolism, and cell growth.


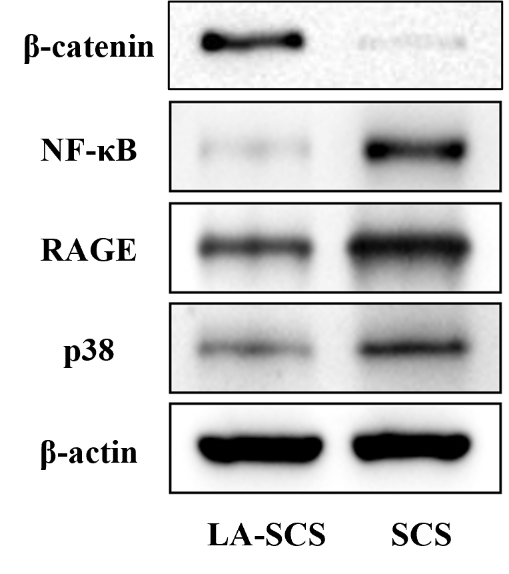


**Figure S9. Western Blot analysis of LA-SCS and SCS groups.** The β-catenin (the core effector of Wnt signal pathway), NF-κB (the downstream inflammatory effector of AGE-RAGE signal pathway), RAGE (the core receptor of AGE-RAGE pathway), and p38 (the key effector of MAPK signal pathway) was significantly regulated in the LA-SCS group, consistent with the result of RNA-seq.


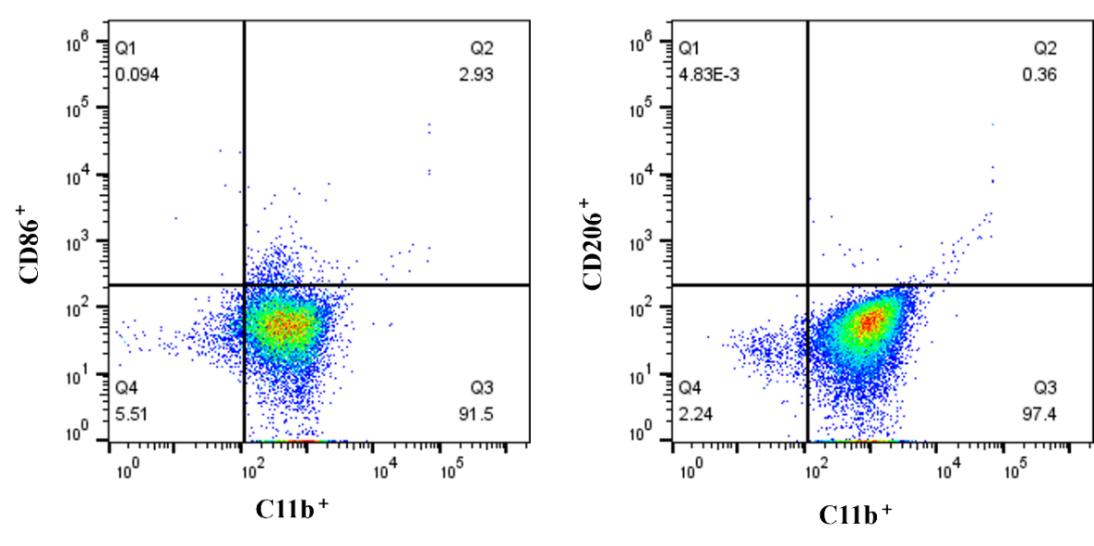


**Figure S10. Flow cytometric identification of macrophages.** The results demonstrated successful isolation of macrophages, with a high proportion of CD11b⁺ positive cells. Additionally, the expression levels of CD86⁺ and CD206⁺ were relatively low, indicating that only a small number of macrophages exhibited a polarized phenotype. n = 5.


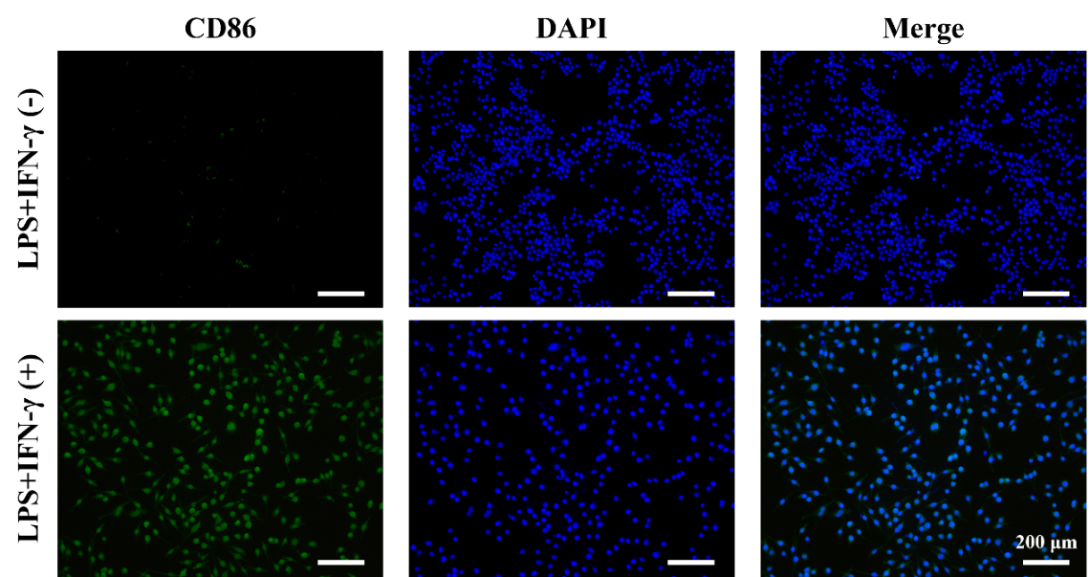


**Figure S11. CD86 immunofluorescence staining for evaluating the induction efficacy of M1 macrophages.** In the LPS+IFN-γ (-) group, green fluorescence was extremely faint, while the LPS+IFN-γ (+) group displayed prominent CD86-associated green fluorescence, confirming the successful induction of M1 polarization.


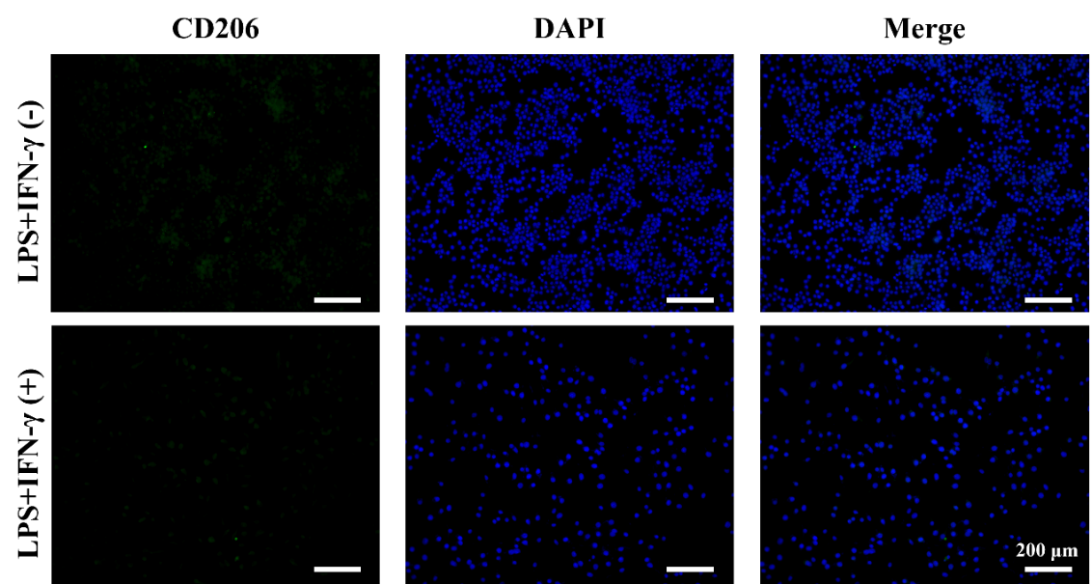


**Figure S12. CD206 immunofluorescence staining for evaluating the efficacy of M1 macrophage induction.** Green fluorescence was extremely faint in both the LPS+IFN-γ (-) and LPS+IFN-γ (+) group, indicating that macrophages did not polarize toward the M2 phenotype. This indirectly confirmed the specificity of M1 polarization induction.


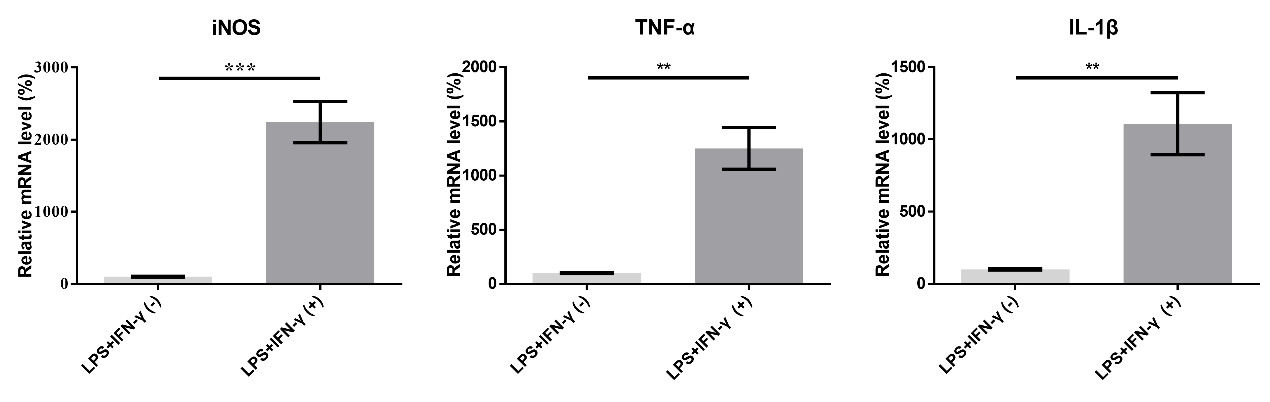


**Figure S13. Evaluation of gene expression for assessing the efficacy of M1 macrophage induction.** The gene expression levels of iNOS, TNF-α, and IL-1β in the LPS+IFN-γ (+) group were significantly elevated. Independent sample t test was used for statistical analysis; n = 5; All data were depicted as means ± SD; ^**^*p* < 0.01, ^***^*p* < 0.001.


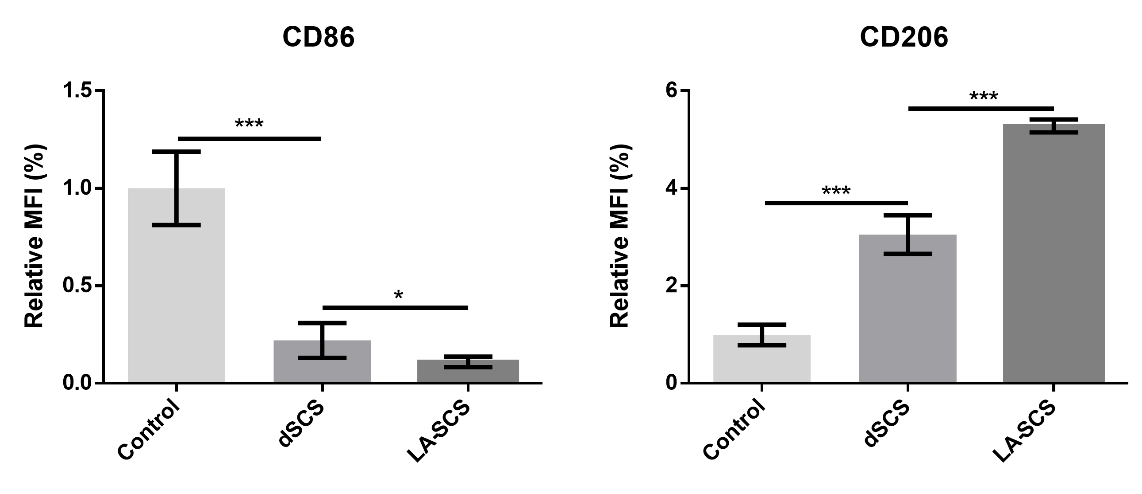


**Figure S14. CD86/CD206 mean fluorescence intensity (MFI).** The MFI results also indicated that the LA-SCS group has the best immunomodulatory ability. Independent sample t test was used for statistical analysis; n = 5; All data were depicted as means ± SD; ^*^*p* < 0.05, ^***^*p* < 0.001.


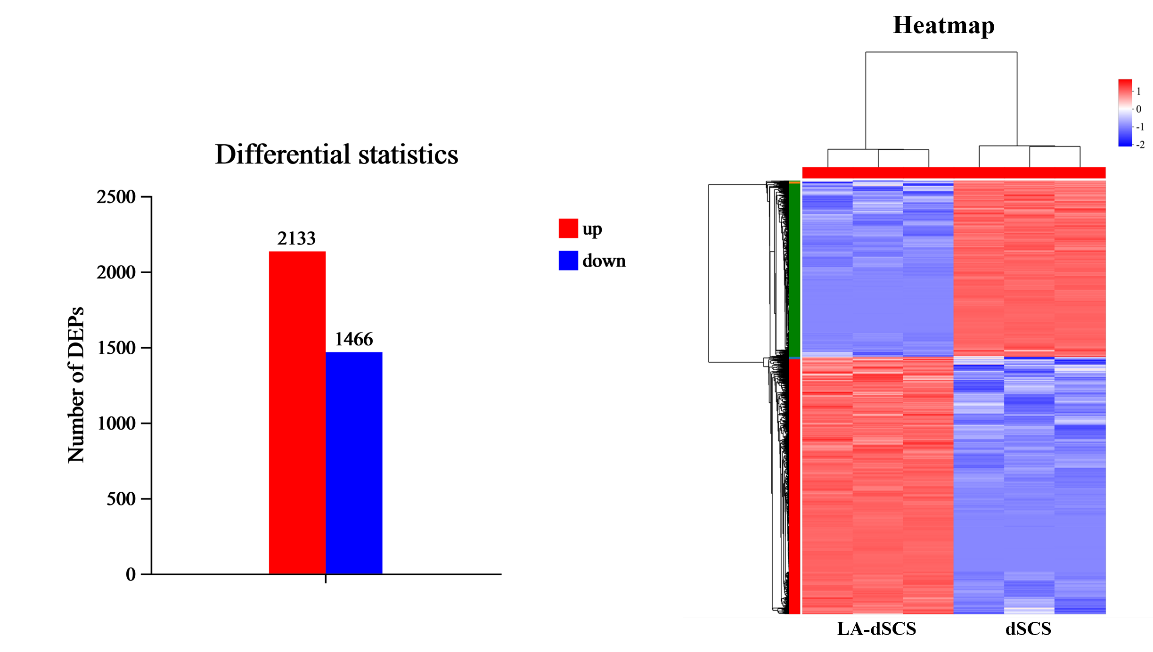


**Figure S15. Quantification of differentially expressed proteins and heatmap analysis of macrophage proteomic data.** The heatmap results revealed distinct differences in protein expression, with samples within each group showing good expression consistency. n = 5.


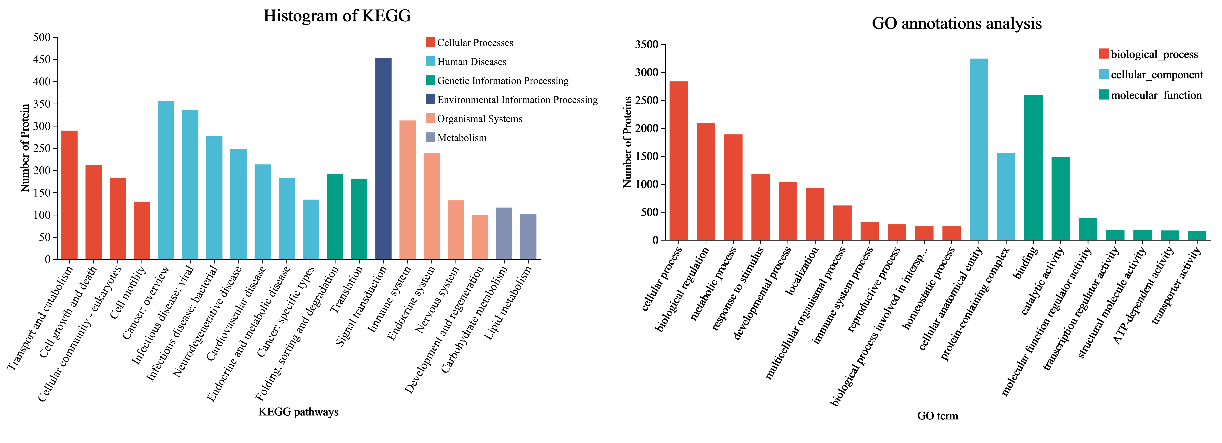


**Figure S16. Functional annotation analysis of proteomics data.** KEGG functional annotation results indicated that a relatively large number of differentially expressed proteins were enriched in categories such as signal transduction, transport, and catabolism. GO annotation analysis revealed alterations in multiple biological processes, including regulation of cellular processes, metabolic processes, response to stimuli, developmental processes, regulation of molecular functions, regulation of transcriptional functions, and ATP-dependent activities. These findings suggested that macrophages undergo comprehensive differential regulation at multiple levels, including molecular, protein, and metabolic levels.


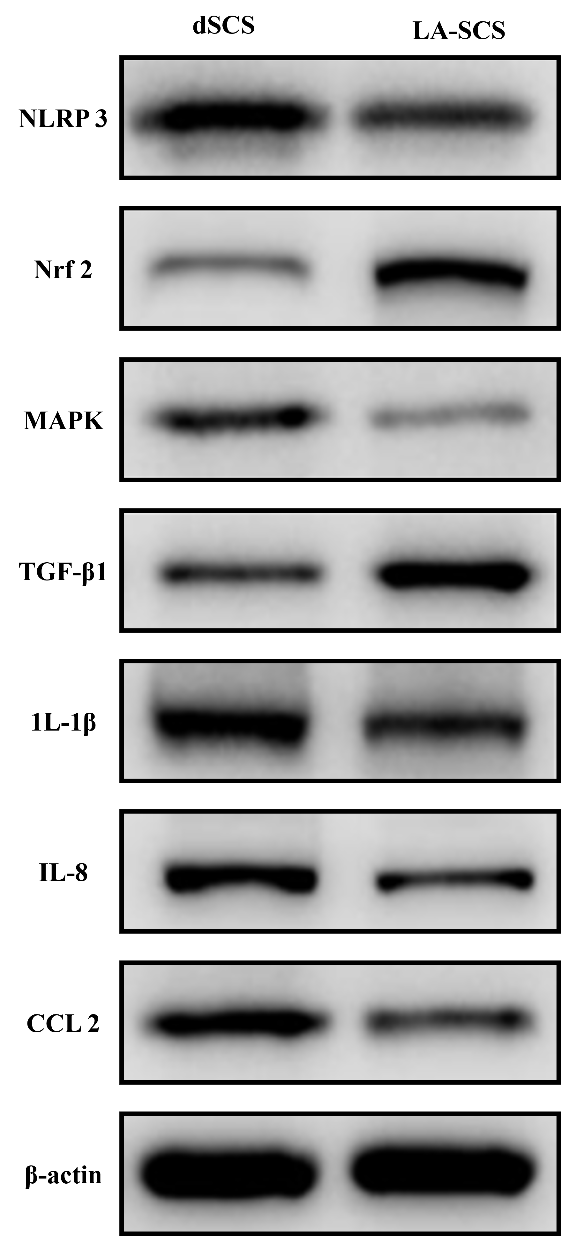


**Figure S17. Western blot analysis of macrophages.** The results demonstrated that macrophages in the LA-dSCS group exhibited lower expression levels of inflammation- and migration-related proteins, which further validated the findings from proteomic analysis.


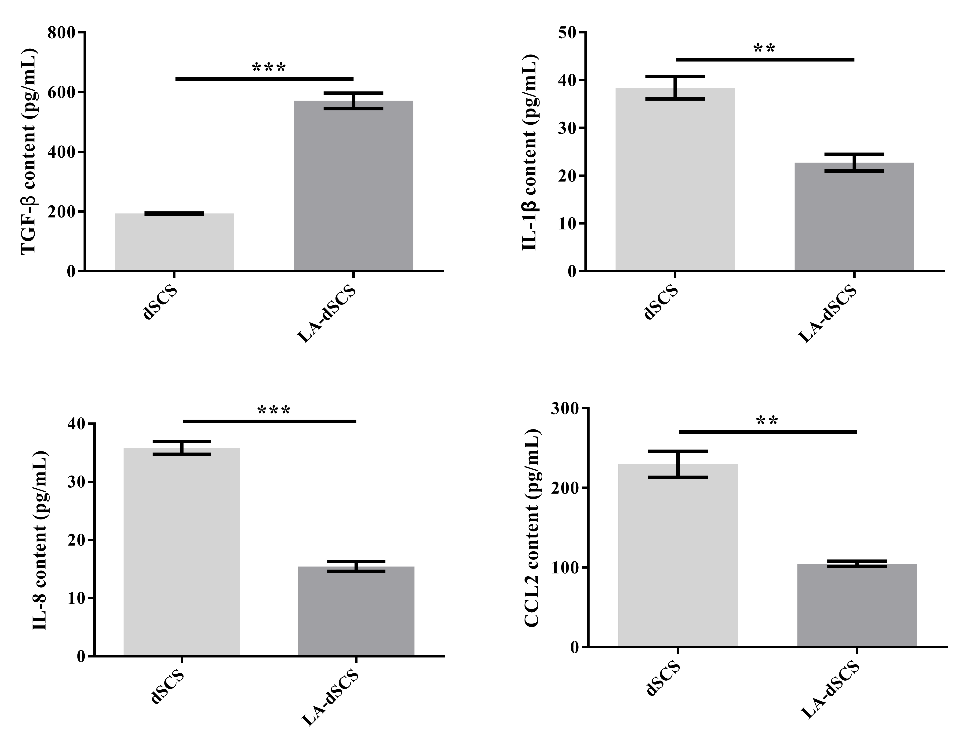


**Figure S18. ELISA analysis of macrophages.** The results revealed that macrophages in the LA-dSCS group exhibited increased expression of the anti-inflammatory factor TGF-β, decreased expression of the pro-inflammatory factor IL-1β, and reduced expression of the migration-related proteins IL-8 and CCL2. These findings further confirmed the results of proteomic analysis. Independent sample t test was used for statistical analysis; n = 5; All data were depicted as means ± SD; ^**^*p* < 0.01, ^***^*p* < 0.001.


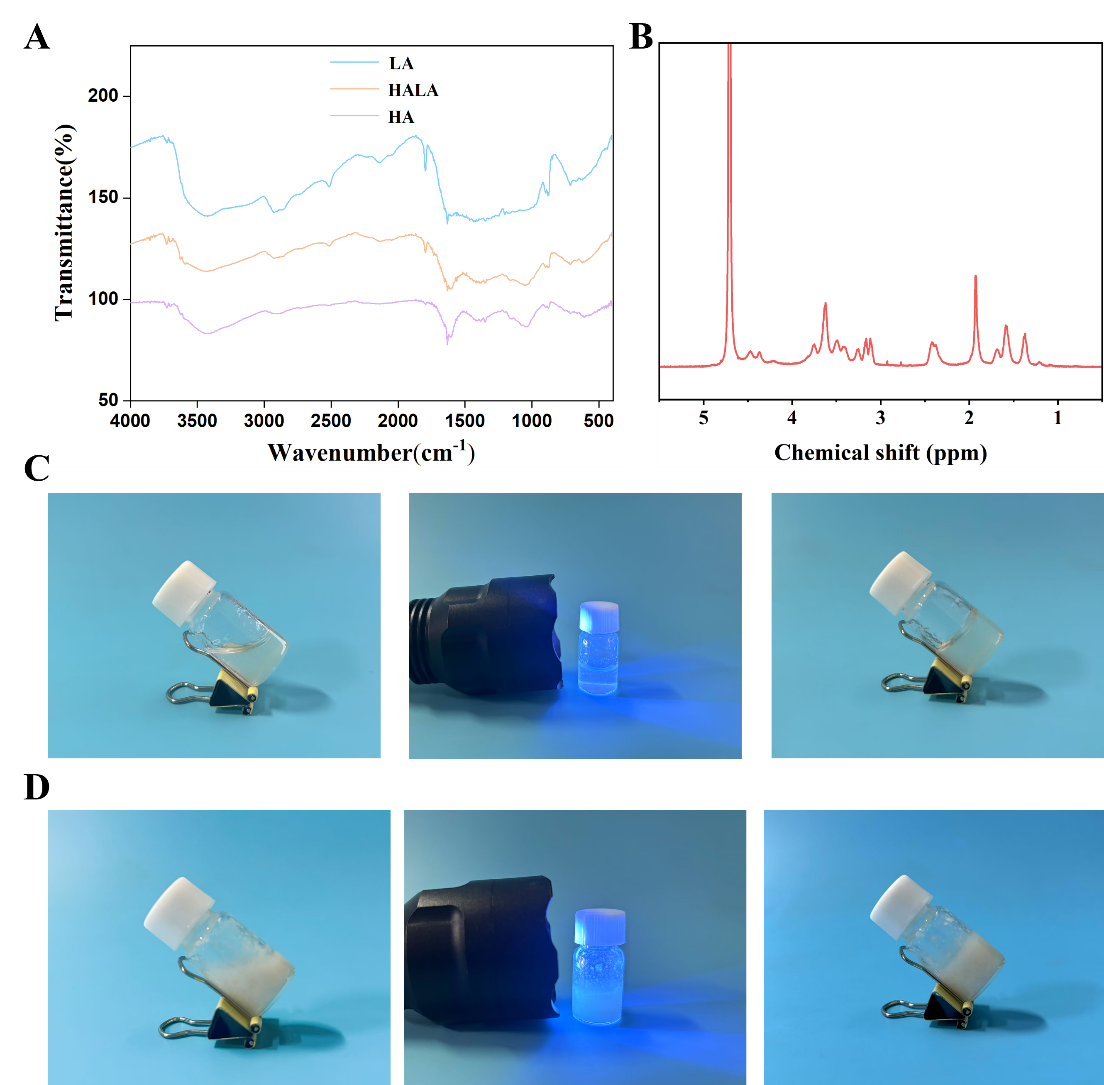


**Figure S19. FTIR, ¹H NMR and photopolymerization capability analysis.** (A, B) FTIR and ¹H NMR analysis confirmed the successful synthesis of HALA. (C) The dithiolane contained in HALA was photosensitive and can be cured upon UV irradiation without additional photoinitiator.


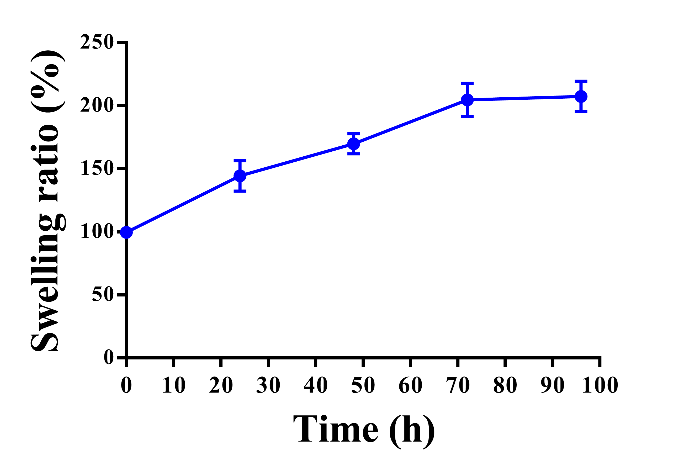


**Figure S20. Quantitative analysis of the swelling of LA-dSCS granular gel.** The swelling results showed that the mass increased rapidly in the first three days and then stabilized afterwards. n = 5; All data were depicted as means ± SD.

**
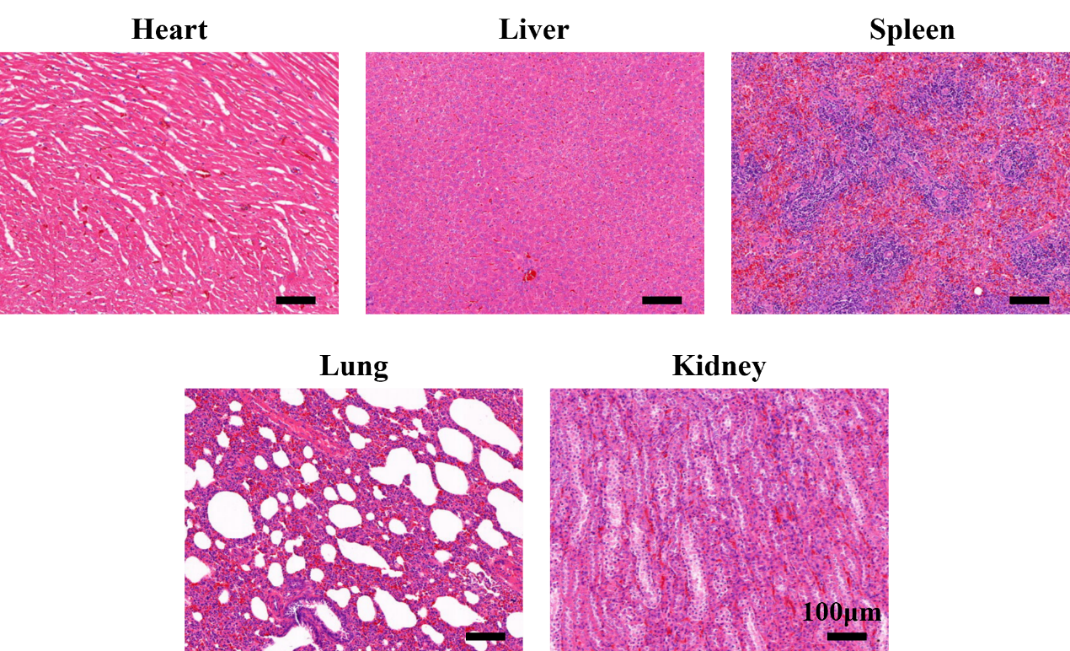
**

**Figure S21. Evaluation of the safety profile of LA-dSCS granular gel.** Four weeks after subcutaneous implantation in rats, HE staining results demonstrated no significant histopathological abnormalities in major organs including the heart, liver, spleen, lungs, and kidneys. These findings indicated that the hydrogel possesses favorable biosafety.


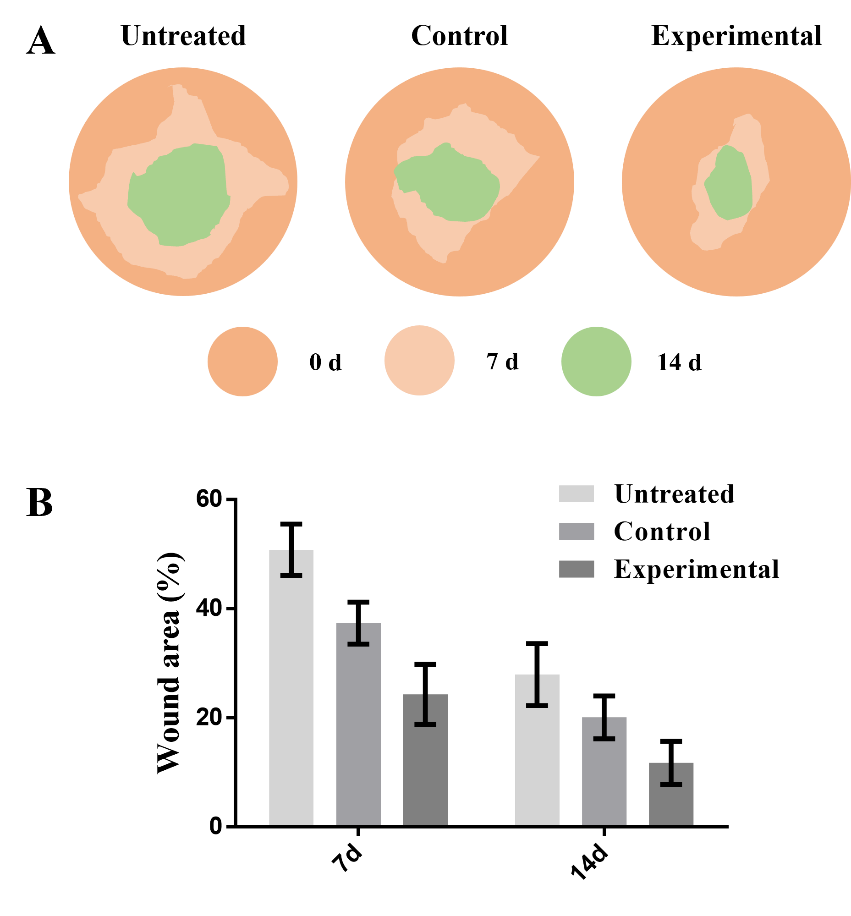


**Figure S22. Quantification of wound healing rate.** (A) The simulated diagram of wound healing. (B) The quantification of wound residual area rate. n = 5; All data were depicted as means ± SD.

**
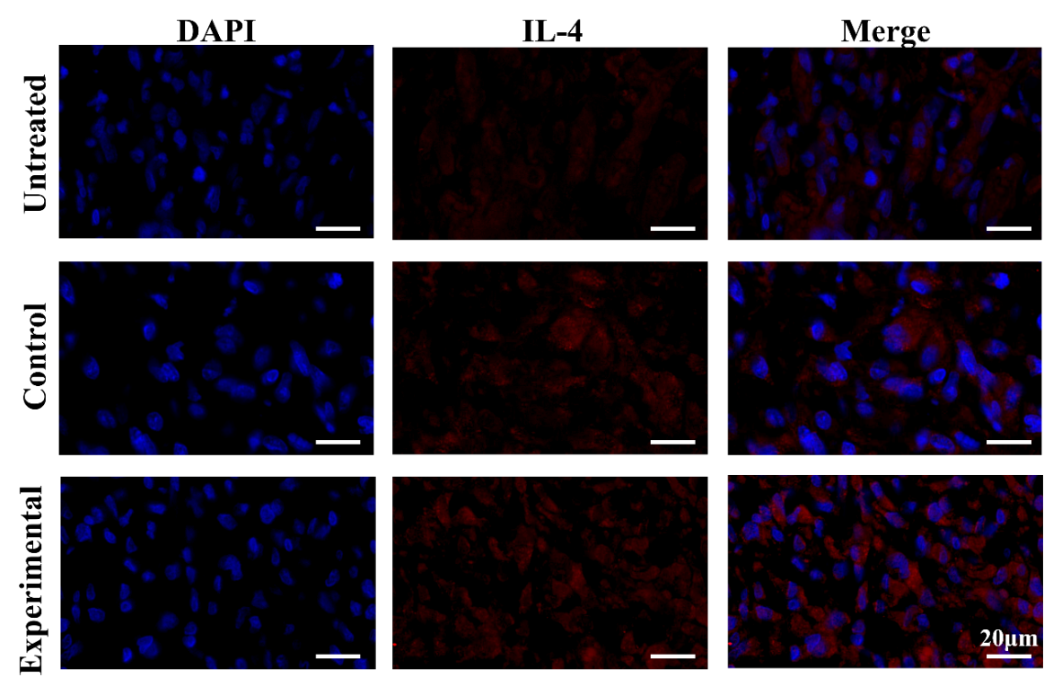
**

**Figure S23. Evaluation of IL-4 inflammatory expression in wound repair.** The results demonstrated that the Experimental group exhibited the highest IL-4 protein expression, indicating the optimal anti-inflammatory capacity.

**
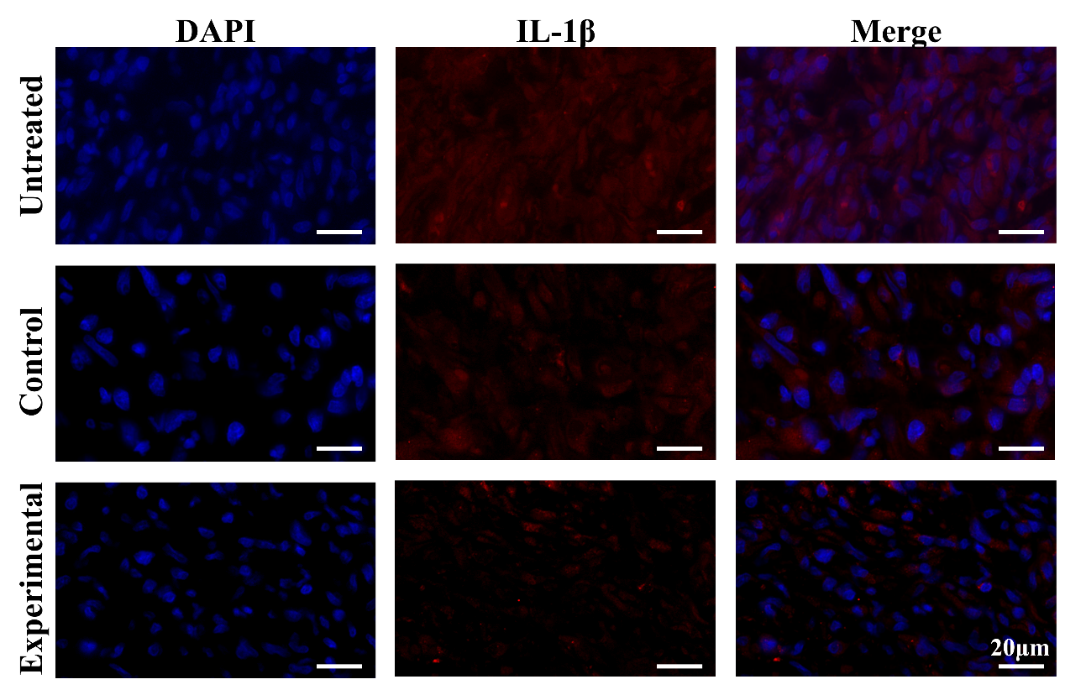
**

**Figure S24. Evaluation of IL-1β inflammatory expression in wound repair.** The results demonstrated that the Experimental group exhibited the lowest IL-1β protein expression, confirming its optimal anti-inflammatory capacity.


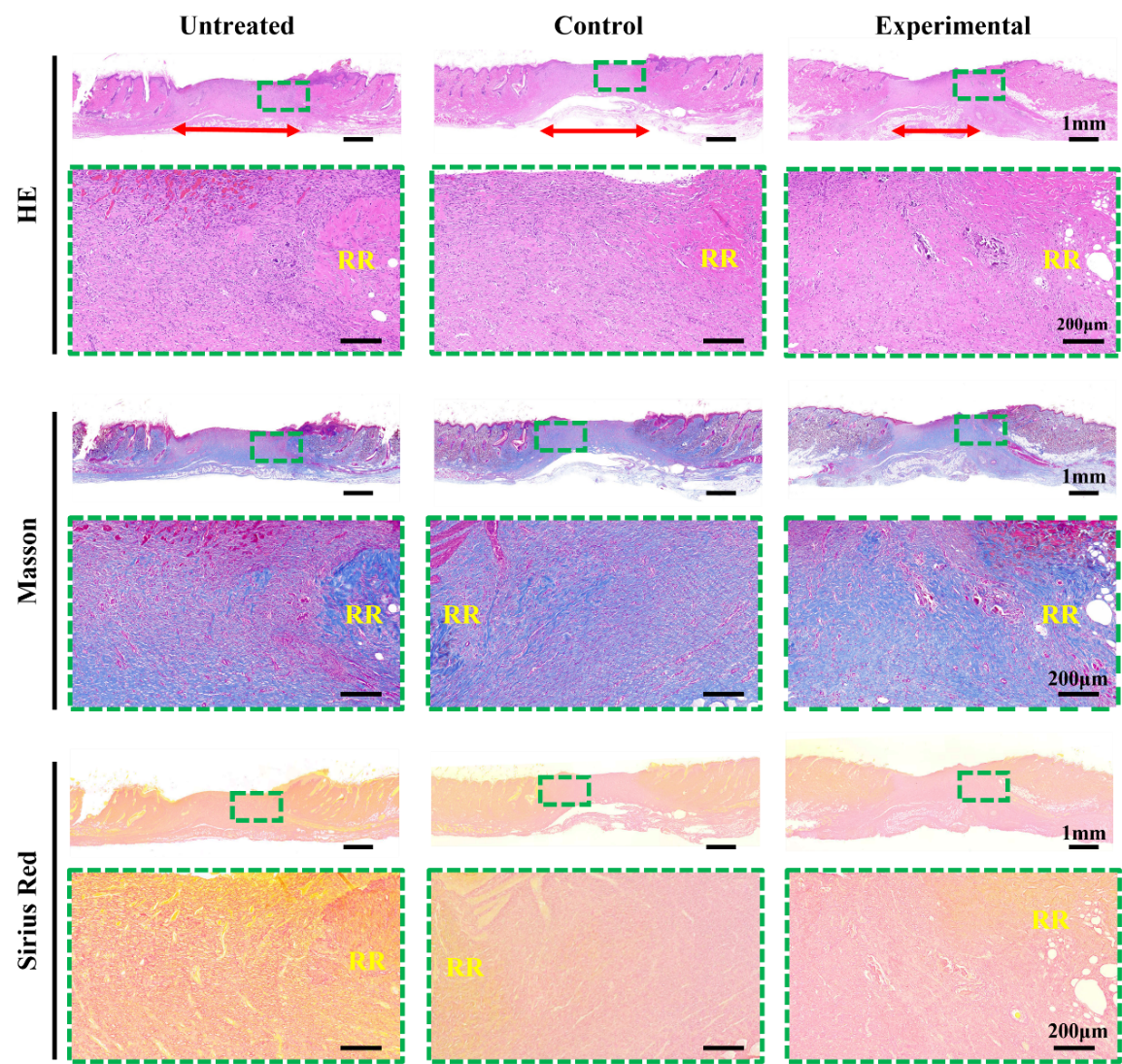


**Figure S25. Evaluation of wound repair efficacy at 7 days.** The results of the current study demonstrated that the Experimental group exhibited the fastest wound closure rate and collagen deposition capacity.

**
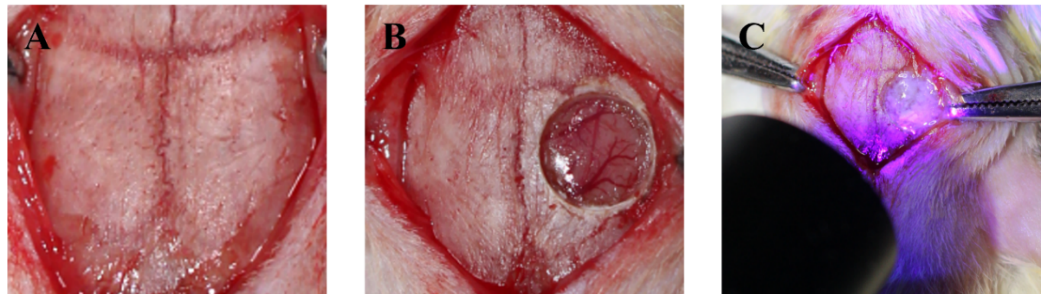
**

**Figure S26. Establishment of skull defect model.** (A) Photograph of rat skull prior to model preparation. (B) Photograph of rat skull after successful model preparation. (C) Photograph showed the injection of LA-dSCS granular gel composite into the defect area in the Experimental group after model preparation.


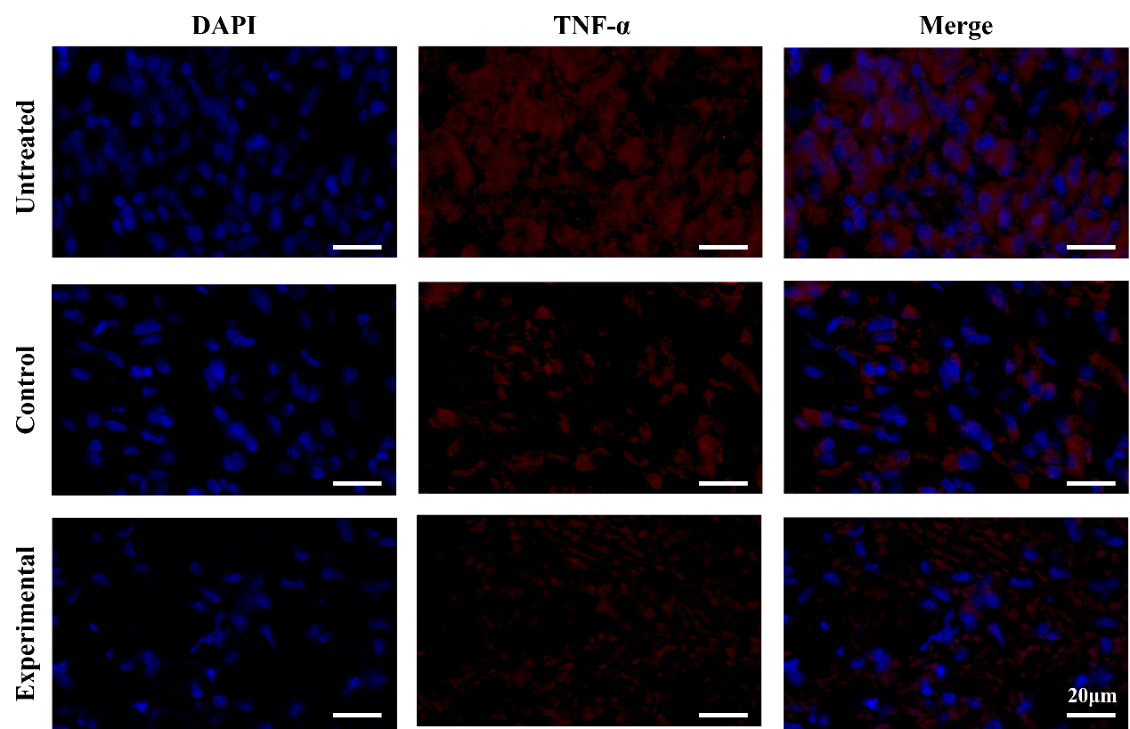


**Figure S27. Evaluation of TNF-α inflammatory expression in skull repair.** The results demonstrated that the Experimental group exhibited the lowest TNF-α expression, confirming its optimal anti-inflammatory capacity.


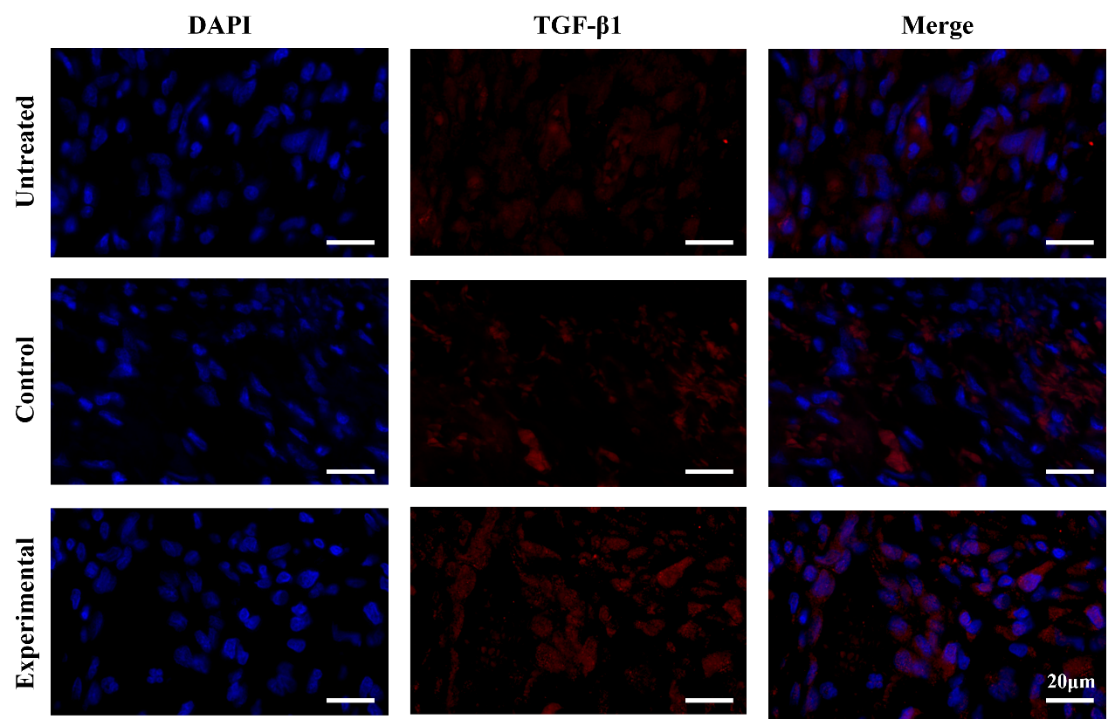


**Figure S28. Evaluation of TGF-β inflammatory expression in skull repair.** The results demonstrated that the Experimental group exhibited the highest TGF-β fluorescent expression, further confirming its optimal anti-inflammatory capacity.

**Table S1. Primer Sequences**

| Gene | Forward Primer (5' to 3') | Reverse Primer (5' to 3') |
| --- | --- | --- |
| NANOG | CTTGCCGTTGGGCTGACAT | GCTTTAGCTTGGGATTGCTAGAA |
| OCT4 | AAGAGGATCACCTTGGGGTACA | CACCAGGGTCTCCGATTTG |
| SOX2 | CTTCGCAGGGAGTTCTCAAAA | TTCCTTCCTTGTCTGTAACGGTC |
| TNF-α | CCACTC TGACCCCTT TACTC | GCCATA ATCCCC TTTCTA AGT |
| BAX | GACACCTGAGCTGACCTTGG | GAGGAAGTCCAGTGTCCAGC |
| HIF-1α | CAAAGACAATAGCTTTGCAGAATG | ACGGTCACCTGGTTGCTG |
| VEGF | CGACAGAAGGGGAGCAGAAA | GCTGGCTTTGGTGAGGTTTG |
| iNOS | CAGAGGACCCAGAGACAAGC | TGCTGAAACATTTCCTGTGC |
| TNF-α | CCACTC TGACCCCTT TACTC | GCCATA ATCCCC TTTCTA AGT |
| IL-1β | AATCTCACAGCAGCATCTCGACAAG | TCCACGGGCAAGACATAGGTAGC |
| BMP-2 | GTGAGGATTAGCAGGTCTTTGC | CTCGTTTGTGGAGTGGATGTC |
| OCN | TGCAAAGCCCAGCGACTCT | TTGAGCTCACACACCTCCCTGT |
| Runx2 | CAAGTGGCCAGGTTCAACGA | GGGACCGTCCACTGTCACTTTAATA |
| GAPDH | AAGGTCGGAGTCAACGGATTT | AGATGATGACCCTTTTGGCTC |
